# Supplementary material for: ImmTOR nanoparticles enhance AAV transgene expression after initial and repeat dosing in a mouse model of methylmalonic acidemia
Source: Mol Ther Methods Clin Dev. 2021 Jul 16;22:279–92. doi: 10.1016/j.omtm.2021.06.015 (PMC8399083; doi:10.1016/j.omtm.2021.06.015)
Supplement: Document S1. Table S1 and Figures S1–S8 [file mmc1.pdf]

OMTM, Volume *22*

Supplemental information

ImmTOR nanoparticles enhance AAV transgene expression after initial and repeat dosing in a mouse model of methylmalonic acidemia

Petr O. Ilyinskii, Alicia M. Michaud, Gina L. Rizzo, Christopher J. Roy, Sheldon S. Leung, Stephanie L. Elkins, Teresa Capela, Aparajita Chowdhury, Lina Li, Randy J. Chandler, Irini Manoli, Eva Andres-Mateos, Lloyd P.M. Johnston, Luk H. Vandenberghe, Charles P. Venditti, and Takashi Kei Kishimoto

Supplementary Materials

Supplementary Table S1. Absolute levels of plasma MMA (pMMA) at 14 days after treatment with Anc80-MMUT, ImmTOR and dexamethasone (Dex-P). Averages ± SD are shown for each group. Number of subjects in each group is shown in parentheses.

Treatment	Anc80-MMUT (15)	Anc80-MMUT (15)	Anc80-MMUT (12)	Anc80-MMUT (7)	Anc80-MMUT (7)
AAV	2.5E12 vg/kg	2.5E12 vg/kg	2.5E12 vg/kg	2.5E12 vg/kg	2.5E12 vg/kg
ImmTOR	None	100 µg	300 µg	None	300 µg
Dexp-P	None	None	None	10 mg/kg	10 mg/kg
pMMA	228 ± 98 µM	127 ± 39 µM	159 ± 80 µM	246 ± 107 µM	100 ± 23 µM

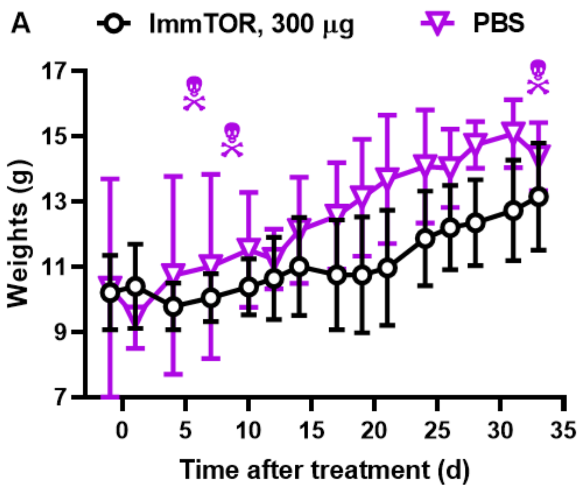

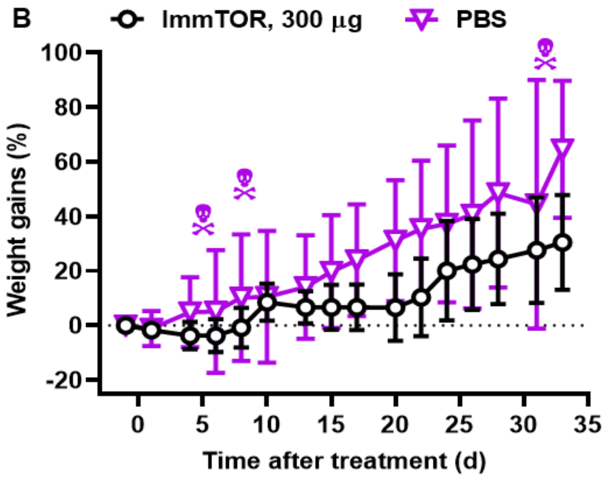

Supplementary Figure S1. Weight gains in MCK mice treated with ImmTOR or mock-treated. Absolute weights (**A**) or weight gains in % vs. pre-injection weight (**B**) are shown with times of mouse deaths in mock-treated group indicated by crossbones sign.

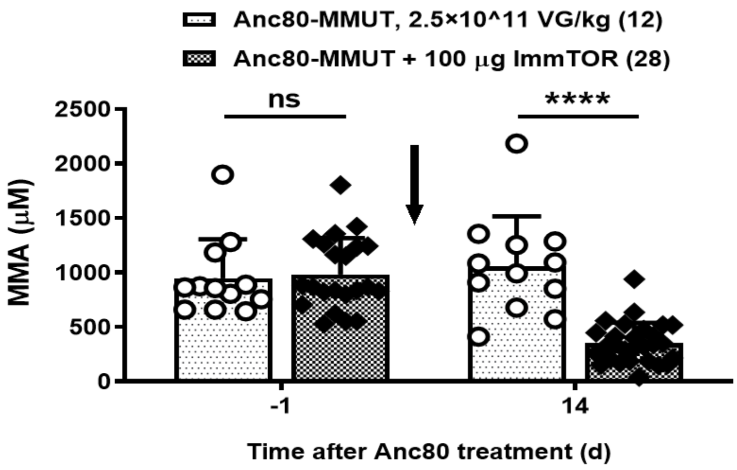

Supplementary Figure S2. ImmTOR provides added benefit at subtherapeutic doses of Anc80-MMUT. *Mmut*$^{-/-}$*;* Tg$^{\text{INS-MCK-}Mmut}$ mice at 14 days of age were treated once with Anc80-MMUT (2.5×10^{11} vg/kg) either alone or combined with ImmTOR (100 µg). Plasma methylmalonic acid (pMMA) levels before the treatment (day -1) and at 14 days after a single Anc80-MMUT administration are shown with numbers of mice in each group indicated in parentheses. Statistical significance is indicated (ns – not significant, **** – p <0.0001).

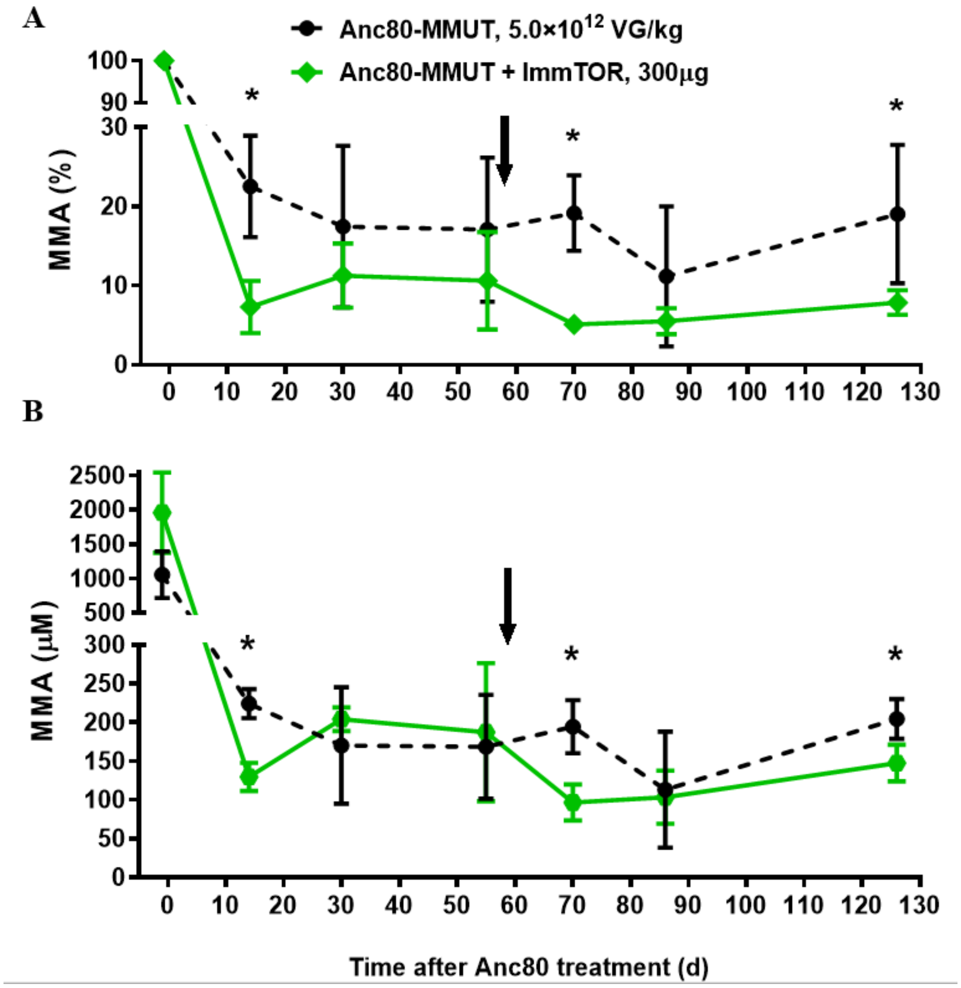

Supplementary Figure S3. Co-administration of high therapeutic dose of Anc80-MMUT with ImmTOR elevates transgene activity. Anc80-MMUT (5.0×10^{12} vg/kg) alone or combined with 300 µg of ImmTOR was injected twice on days 0 and 56 in groups of 3-4 mice. Plasma MMA levels are shown for the duration of the study (126 days after initial treatment) as % vs. pre-treatment levels as 100% (**A**) or in absolute numbers (**B**). Time-points with statistically significant (* – $p<0.05$) differences vs. group not receiving ImmTOR are indicated. Day 56 repeat injection is indicated by arrow. Averages with SD are shown.

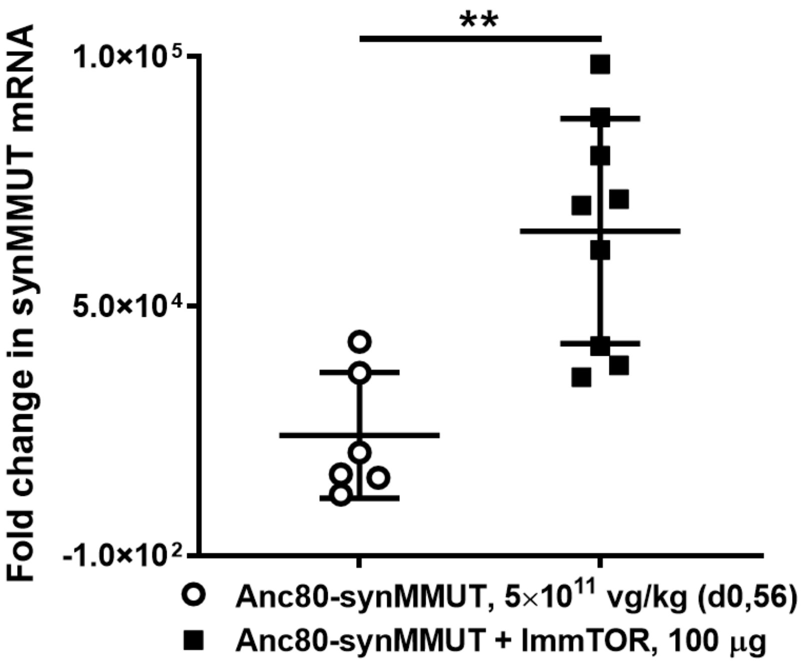

Supplementary Figure S4. AAV-driven *MMUT* mRNA expression after repeated injections with Anc80-MMUT combined with ImmTOR. *synMMUT* mRNA levels in livers of wild-type C57BL/6 female mice after two (days 0 and 56) injections with Anc80-synMMUT with or without 100 µg of ImmTOR as measured by fold increase (GAPDH-normalized) over untreated baseline 43 days after repeat vector dose (day 99 after initial treatment). Statistical significance (** – p<0.01) is shown. Averages with SD are shown.

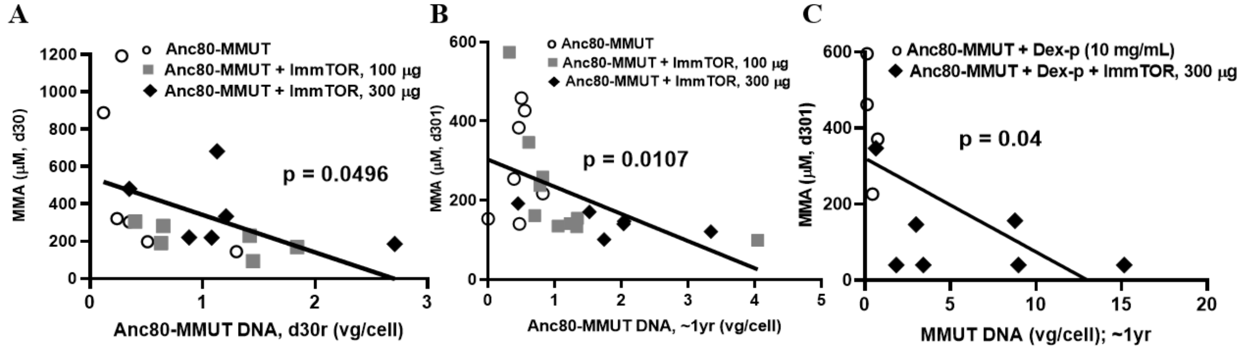

Supplementary Figure S5. Inverse correlation of short-term and long-term AAV transduction efficacy and serum levels of methylmalonic acid in individual studies. **A.** Viral DNA copy numbers and serum levels of methylmalonic acid have been measured on day 30 following a single injection with Anc80-MMUT alone or combined with 100 or 300 µg ImmTOR. Data shown in Figs. 1B and 2A are used for analysis. **B, C.** Viral DNA copy numbers and serum levels of methylmalonic acid have been measured 1 year after two doses of Anc80-MMUT administered alone or combined with 100 or 300 µg ImmTOR. Data from Figs. 4A and 4C (**B**) or from Figs.

5A and 5C (**C**) are used for analysis. P value of correlation is shown for each graph.

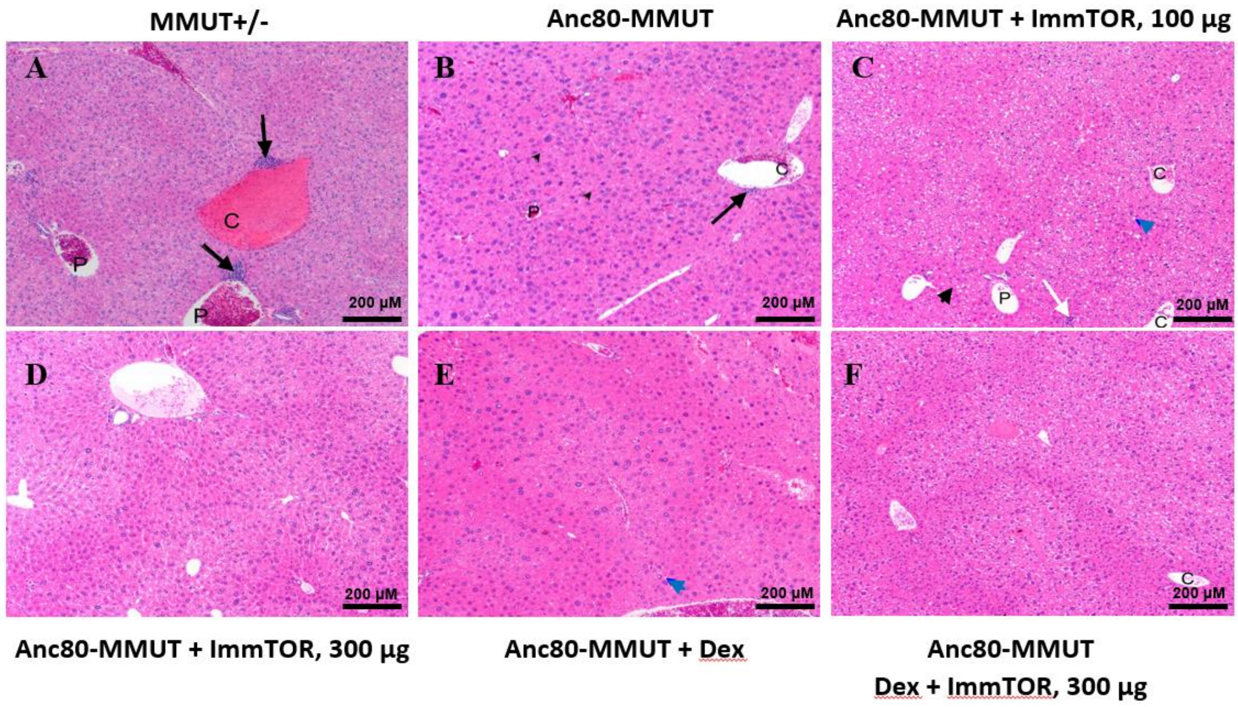

Supplementary Figure S6. Long-term alleviation of hepatic pathology by Anc80-MMUT and ImmTOR. Representative photomicrographs of liver sections from heterozygous *Mmut*$^{+/-}$ (**A**),

and Anc80-MMUT-treated *Mmut*$^{-/-}$*;* Tg$^{INS\text{-}MCK\text{-}Mmut}$ mice (**B-F**) are shown (40×). Anc80-MMUT (days 0 and 56) was used alone (B) or combined with 100 µg (**C**) or 300 µg (**D**) of ImmTOR, combined with dexamethasone (**E**) or dexamethasone and 300 µg ImmTOR (**F**). Hematoxylin and eosin staining of liver sections from 1-year old mice are shown, scale bars: 200 µm. Heterozygous control (**A**) shows small aggregates of perivascular mononuclear cells (black arrows) adjacent to both portal tracts (P) and central veins (C). Livers from mice treated Anc80-MMUT (**B**, **E**) showed minimal mononuclear cell infiltration (black arrow) accompanied by a few necrotic hepatocytes as well as low numbers of hepatocytes with intracytoplasmic inclusions (black arrowheads) or vacuoles (blue arrowheads). Mice co-treated with low dose ImmTOR (**C**) shown minimal, random mixed cell inflammation (white arrows), hepatocyte vacuolization (blue arrowheads), and hepatocyte intracytoplasmic inclusions (black arrowheads), while in mice co-treated with high dose ImmTOR (**D**, **F**) histologic findings were minimal to absent.

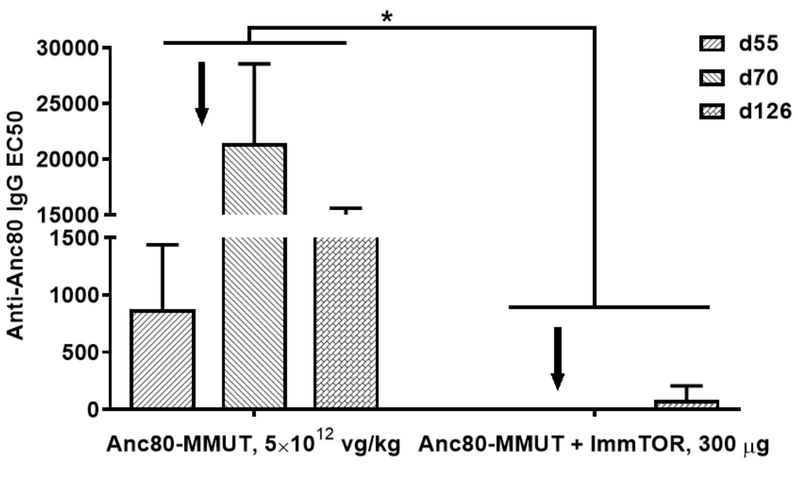

Supplementary Figure S7. Co-administration of high therapeutic dose of Anc80-MMUT with ImmTOR prevents IgG antibody formation. Anc80-MMUT (5.0×10^{12} vg/kg) alone or combined with 300 µg of ImmTOR was injected twice on days 0 and 56 in groups of 3-4 *Mmut*$^{-/-}$

; Tg$^{INS\text{-}MCK\text{-}\mathit{Mmut}}$ mice. EC_{50} of Anc80 IgG in serum was measured at times indicated. Time-points with statistically significant (* – $p<0.05$) differences between groups are indicated. Day 56 repeat injection is indicated by arrow. Averages with SD are shown.

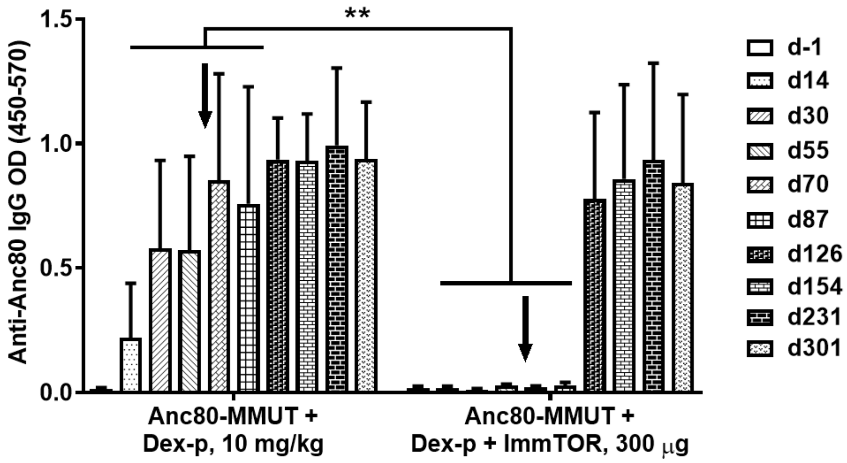

Supplementary Figure S8. Co-administration of high therapeutic dose of Anc80-MMUT with ImmTOR and dexamethasone prevents IgG antibody formation, while dexamethasone alone is ineffective. Anc80-MMUT (2.5×10^{12} vg/kg) alone or combined with 300 µg of ImmTOR was injected twice on days 0 and 56 in groups of 5-7 *Mmut*$^{-/-}$; Tg$^{INS\text{-}MCK\text{-}\mathit{Mmut}}$ mice (see Fig. 5). Both groups also received 10 mg/ml dexamethasone concurrently with Anc80 inoculation. Anc80 IgG in serum was measured at times indicated and presented as top OD. Time-points with statistically significant (** – $p<0.01$) differences between groups receiving and not receiving ImmTOR are indicated. Day 56 repeat injection is indicated by arrow. Averages with SD are shown.
