# Supplementary material for: ImmTOR nanoparticles enhance AAV transgene expression after initial and repeat dosing in a mouse model of methylmalonic acidemia
Source: Mol Ther Methods Clin Dev. 2021 Jul 16;22:279–92. doi: 10.1016/j.omtm.2021.06.015 (PMC8399083; doi:10.1016/j.omtm.2021.06.015)
Supplement: Document S2. Article plus supplemental information [file mmc2.pdf]

Molecular Therapy
Methods & Clinical Development
Original Article

ImmTOR nanoparticles enhance AAV transgene expression after initial and repeat dosing in a mouse model of methylmalonic acidemia

Petr O. Ilyinskii,[1] Alicia M. Michaud,[1] Gina L. Rizzo,[1] Christopher J. Roy,[1] Sheldon S. Leung,[1] Stephanie L. Elkins,[1] Teresa Capela,[1] Aparajita Chowdhury,[1] Lina Li,[2] Randy J. Chandler,[2] Irini Manoli,[2] Eva Andres-Mateos,[3,4] Lloyd P.M. Johnston,[1] Luk H. Vandenberghe,[3] Charles P. Venditti,[2] and Takashi Kei Kishimoto[1]

[1]Selecta Biosciences, Watertown, MA, USA; [2]National Human Genome Research Institute, National Institutes of Health, Bethesda, MD, USA; [3]Massachusetts Eye and Ear Infirmary, Harvard Medical School, Boston, MA, USA

A major barrier to adeno-associated virus (AAV) gene therapy is the inability to re-dose patients due to formation of vector-induced neutralizing antibodies (Nabs). Tolerogenic nanoparticles encapsulating rapamycin (ImmTOR) provide long-term and specific suppression of adaptive immune responses, allowing for vector re-dosing. Moreover, co-administration of hepatotropic AAV vectors and ImmTOR leads to an increase of transgene expression even after the first dose. ImmTOR and AAV Anc80 encoding the methylmalonyl-coenzyme A (CoA) mutase (MMUT) combination was tested in a mouse model of methylmalonic acidemia, a disease caused by mutations in the *MMUT* gene. Repeated co-administration of Anc80 and ImmTOR was well tolerated and led to nearly complete inhibition of immunoglobulin (Ig)G antibodies to the Anc80 capsid. A more profound decrease of plasma levels of the key toxic metabolite, plasma methylmalonic acid (pMMA), and disease biomarker, fibroblast growth factor 21 (FGF21), was observed after treatment with the ImmTOR and Anc80-MMUT combination. In addition, there were higher numbers of viral genomes per cell (vg/cell) and increased transgene expression when ImmTOR was co-administered with Anc80-MMUT. These effects were dose-dependent, with the higher doses of ImmTOR providing higher vg/cell and mRNA levels, and an improved biomarker response. Combining of ImmTOR and AAV can not only block the IgG response against capsid, but it also appears to potentiate transduction and enhance therapeutic transgene expression in the mouse model.

INTRODUCTION

Isolated methylmalonic acidemia (IMMA) is a rare and heterogeneous autosomal recessive metabolic disorder affecting the metabolism of branched-chain amino acids, odd-chain fatty acids, and cholesterol. Patients with IMMA massively accrete "toxic" metabolites, and they share a common clinical phenotype characterized by metabolic decompensations and early mortality.[1–5] IMMA is most frequently caused by mutations in the gene encoding the mitochondrial localized enzyme, methylmalonyl-coenzyme A (CoA) mutase (MMUT). This enzyme catalyzes the isomerization of L-methylmalonyl-CoA into succinyl-CoA. Reduced activity of MMUT result in the production of plasma methylmalonic acid (pMMA) that can be measured in plasma and serum as a biomarker of disease. Patients with the most severe form of MMUT deficiency (*MMUT*^{0}) can experience severe ketoacidosis and hyperammonemia, leading to irreversible cognitive deficits and high mortality in early childhood.[6–9] The main treatment of IMMA, first established in the 1960s,[10,11] is through dietary restriction of offensive amino acids[12,13] but is insufficient to prevent disease progression, as well as metabolic decompensations, which can be lethal. Despite substantial metabolite production in extrahepatic tissues,[14] liver transplantation, when successful, leads to remarkable metabolic stability of the recipient,[15–20] and it provides evidence that the liver must be the major therapeutic target organ for IMMA. Indeed, clinical observations have been supported by both transgenic animal modeling[21] and proof-of-concept gene therapy studies in IMMA mice[9,22] that unequivocally demonstrate the requirement to augment hepatic MMUT activity for therapeutic benefit in IMMA.

Adeno-associated virus (AAV)-mediated liver-directed gene therapy is emerging as a clinically relevant treatment option for the treatment of inborn errors of metabolism, especially those with hepatic manifestations. During the past decade, numerous studies in preclinical models of IMMA have demonstrated that the neonatal administration of Ad5, AAV2, AAV8, or AAV9 vectors expressing the *MMUT* gene under either the transcriptional control of both ubiquitous and liver-specific promoters could rescue *Mmut*$^{-/-}$ mice, which otherwise would die in the first days after birth.[9,14,22–24] Most recently, a viable but severe mouse model of IMMA designed to recapitulate the hepatic manifestations of the disease was created by

Received 7 June 2020; accepted 30 June 2021;
https://doi.org/10.1016/j.omtm.2021.06.015.

[4]Present address: Akouos, Boston, MA

Correspondence: Takashi Kei Kishimoto, Selecta Biosciences, 65 Grove Street, Watertown, MA 02472, USA.
E-mail: kkishimoto@selectabio.com

This is an open access article under the CC BY license (http://creativecommons.org/licenses/by/4.0/).

transgenic expression of MMUT under the muscle-specific murine creatine kinase (MCK) promoter in *Mmut*$^{-/-}$ mice (*Mmut*$^{-/-}$; Tg$^{\text{INS-MCK-}\textit{Mmut}}$, referred to herein as MCK-MMUT mice). MCK-MMUT mice show increased survival, but they manifest the cardinal features of IMMA, including growth impairment, metabolic fragility, susceptibility to dietary and environmental stress, hepatorenal mitochondrial pathology, and massively elevated levels of pMMA, similar to what is observed in severely affected IMMA pediatric patients.[9] Liver-directed gene therapy of MCK-MMUT mice corrects the hepatic metabolic defect, as evidenced by weight gain, increased propionate oxidation, and decreased levels of plasma fibroblast growth factor 21 (FGF21), a marker of mitochondrial dysfunction.[5,9,25,26]

Gene therapy for severely affected IMMA patients would be most effective in infants or young children to prevent recurrent metabolic ketoacidosis with intercurrent illnesses leading to irreversible neurological sequelae, improve protein tolerance and growth, and possibly delay the onset of chronic renal disease and other multisystem complications. Due to the non-replicating nature of recombinant AAV vectors, a major concern for gene therapy in pediatric patients is vector degradation as well as its dilution as the child grows, resulting in possible loss of therapeutic benefit.[27] An intrinsic limitation of AAV in human gene therapy is the formation of high-titer neutralizing antibodies (Nabs) that develop after administration, and subsequently prevent re-dosing. We have previously described the development of tolerogenic nanoparticles encapsulating rapamycin (ImmTOR or SVP-rapamycin), which, when co-administered with AAV-based vectors, provide a dose-dependent and long-term suppression of humoral and T cell responses against AAV, and therefore allow for productive AAV vector re-dosing.[28,29] Moreover, co-administration of liver-tropic AAV vectors and ImmTOR leads to immediate increase of transgene expression even after the first dose, most likely due to elevated uptake by liver cells and rapamycin-induced autophagy.[29–31]

In this study, we evaluated the safety and therapeutic efficacy of an ImmTOR and AAV vector combination in a mouse model of IMMA using a vector pseudoserotyped with Anc80, a rationally engineered AAV capsid.[32] Repeated co-administration of Anc80-MMUT and ImmTOR was well tolerated and led to nearly complete inhibition of the formation of immunoglobulin G (IgG) antibodies to Anc80. A more profound and consistent decrease of pMMA after initial and repeat injections was observed in mice treated with the combination of ImmTOR and Anc80-MMUT than in those treated with Anc80-MMUT alone. Additionally, FGF21 plasma levels after repeat AAV injection remained elevated when ImmTOR was not co-administered, while they were consistently decreased after treatment with AAV and ImmTOR combination. Higher liver viral DNA copy number per cell (viral genomes [vg]/cell) as well as higher hepatic *MMUT* mRNA expression levels were seen in mice injected with the combination of ImmTOR and Anc80-MMUT. These effects were dose-dependent, with higher doses of ImmTOR providing higher vg/cell levels and lower pMMA levels. Similar effects were seen at variable Anc80-MMUT doses, including subtherapeutic and high therapeutic doses.

Collectively, the combination of ImmTOR and hepatotropic AAV-MMUT is a promising approach to mitigate the detrimental impact of *de novo* formed IgG on gene therapy for IMMA and related disorders and should enable repeat AAV vector dosing and may also provide an added benefit by increasing vector transduction and elevating transgene expression at the initial dose.

RESULTS

Therapeutic effects in hypomorphic MCK-MMUT mice after initial Anc80-MMUT treatment

The treatment of MCK-MMUT mice (24–28 days of age) with the therapeutic dose of 2.5×10^{12} vg/kg Anc80-MMUT led to rapid and immediate weight gains in all treatment groups. Initially, animals treated with Anc80-MMUT alone showed more rapid weight gain than did groups treated with Anc80-MMUT + ImmTOR; however, the differences became insignificant by the third week after treatment (Figure 1A). Initial lower weight gain was also observed in controls treated with ImmTOR alone versus the mock-treated group (Figure S1), but it was not significant and skewed by higher mortality in the mock-treated group, which mostly affected lower weight animals, but not the ImmTOR-treated group (3/7 in the mock-treated group versus 0/7 in the ImmTOR-treated group). Consistent with the data reported in the literature,[5,9] initial administration of a therapeutic dose of AAV vector containing the *Mmut* gene resulted in a profound (85%) drop of pMMA at 2 weeks post-treatment. Interestingly, this effect was even more pronounced and statistically different when the AAV vector was co-administered with ImmTOR (Figure 1B). The benefit of ImmTOR co-administration was more apparent at a later (day 30) time point at which pMMA increased ~2-fold in animals treated with Anc80-MMUT alone. The addition of ImmTOR at the time of Anc80-MMUT dosing resulted in a dose-dependent inhibition of the rebound in pMMA levels at day 30 (Figure 1B). Collectively, these results suggest that ImmTOR has a dose-dependent benefit on reducing pMMA levels even after the initial dose of Anc80-MMUT, but prior to vector re-dosing. A similar benefit was observed when ImmTOR was co-administered with a subtherapeutic dose of 2.5×10^{11} vg/kg Anc80-MMUT in 14-day-old juvenile MCK mice. Two weeks after treatment, the pMMA levels in mice receiving Anc80-MMUT combined with ImmTOR were 3-fold lower than in mice treated with same low-dose Anc80-MMUT alone, with the latter essentially being non-therapeutic (Figure S2).

We next evaluated plasma FGF21, a biomarker of mitochondrial dysfunction and a key indicator of IMMA progression both in human patients and mouse models.[9] Plasma FGF21 levels dropped precipitously by day 55 in all treatment groups (average pre-treatment levels of 4,090 pg/mL dropping to 573 pg/mL by day 55; Figure 1C), which was similar to the magnitude of reduction described by Manoli et al.[9] (pre-treatment levels of 4,085 pg/mL dropping to 643 pg/mL by day 60).

The dynamics of anti-AAV IgG development was followed after the initial treatment with Anc80-MMUT alone or combined with ImmTOR (Figure 1D). Anti-capsid IgG was detected as early as day

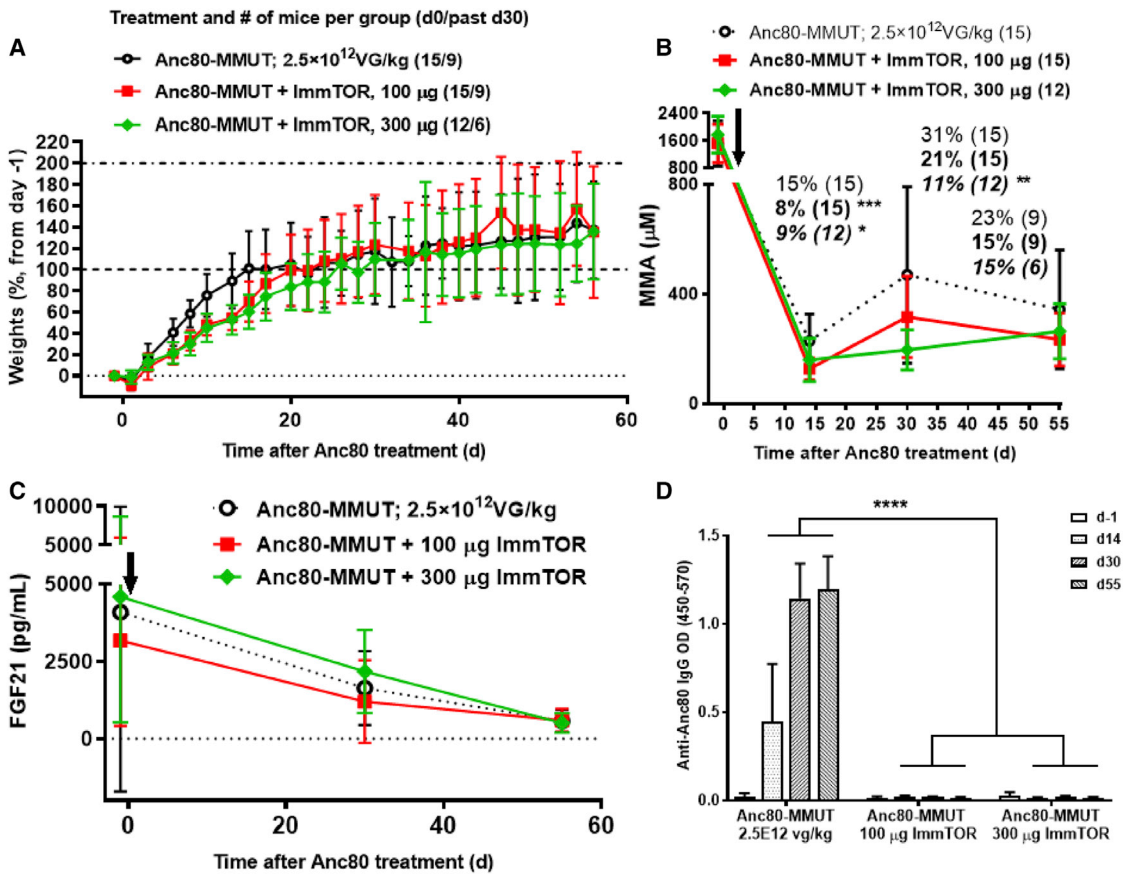

Figure 1. Weight, biomarker, and anti-vector IgG dynamics in *Mmut*$^{-/-}$;Tg$^{INS\text{-}MCK\text{-}Mmut}$ mice after initial treatment with Anc80-MMUT at 2.5 $\times$ 10^{12} vg/kg combined with ImmTOR

(A) Weight gains (in % increase versus pre-injection weight) after initial treatment either with Anc80-MMUT alone or combined with 100 or 300 μg of ImmTOR. Mice were 24–28 days of age at treatment initiation (day 0, indicated by arrow in B and C). (B) Methylmalonic acid concentration in plasma after initial treatment. Relative levels (versus pre-treatment levels as 100%) are also shown for each group at every time point (levels in normal mice were <20 μM). Time points with statistically significant (*p < 0.05, **p < 0.01, ***p < 0.001) differences versus group not receiving ImmTOR are indicated. Number of mice per each group is shown in parentheses (six mice/group were terminated at day 30 for tissue analysis). (C) Dynamics of serum FGF21 levels after initial treatment (levels in normal mice were 207 ± 110 μM). (D) Dynamics of serum IgG antibody response to Anc80-MMUT. Anti-Anc80 IgG is presented as top OD. Averages with SD are shown. Statistical difference between the group treated with Anc80-MUT alone versus those treated with Anc80 combined with ImmTOR is shown for all time points (****p < 0.0001).

14 after initial administration of Anc80 alone and reached substantial quantities by day 30, with all animals in this group having seroconverted. Not a single MCK-MMUT mouse co-treated with ImmTOR seroconverted by day 55, the last time point prior to re-dosing (Figure 1D).

A subset of animals was sacrificed at 30 days after dosing to analyze the efficiency of liver cell transduction after a single injection (Figure 2). Higher levels of transduction were observed in mice treated with a combination of Anc80-MMUT and ImmTOR, although there was no difference between the effects of high and low ImmTOR doses after a single administration. There was also no discernible difference in transduction efficiency between male and female MCK-MMUT mice, as often seen in wild-type mice,[33] probably since they were treated before sexual maturity. A separate study evaluating a higher dose (5E12 vg/kg) of Anc80-MMUT showed superior early MMUT activity (as evidenced by pMMA decrease from baseline) in mice treated with a high dose of ImmTOR, which was maintained after re-dosing, although in this case the impact of ImmTOR was less pronounced (Figure S3).

Overall, there was a clear therapeutic benefit seen after the initial treatment in all experimental groups with ImmTOR providing an additional benefit as evidenced by reduced pMMA levels at days 14 and 30 as well as higher transduction levels at day 30.

Therapeutic effects in hypomorphic MCK-MMUT mice after repeat Anc80-MMUT treatment

A second vector dose of 2.5 $\times$ 10^{12} vg/kg was administered to the remaining mice in all groups on day 56. Mice treated with

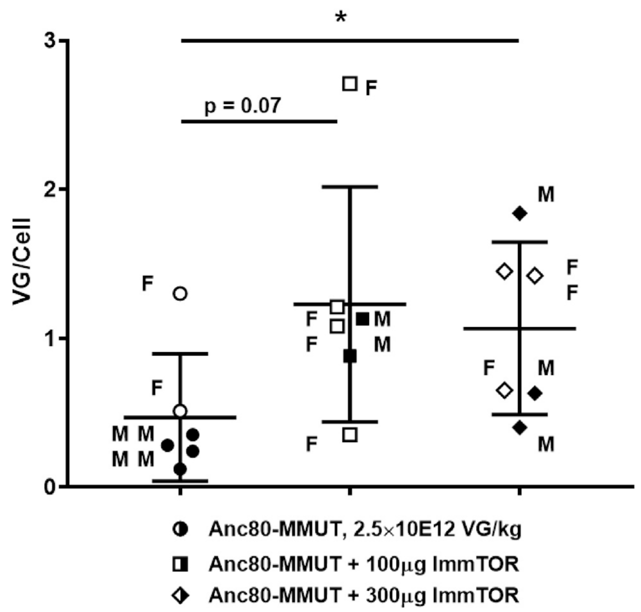

Figure 2. AAV-driven transduction of liver cells of $Mmut^{-/-}$;Tg$^{INS\text{-}MCK\text{-}Mmut}$ mice after initial treatment with Anc80-MMUT combined with ImmTOR

Efficacy of Anc80-MMUT transduction of $Mmut^{-/-}$;Tg$^{INS\text{-}MCK\text{-}Mmut}$ mouse livers with or without ImmTOR (100 or 300 μg) measured as number of viral genome copies per cell (vg/cell) on day 30 after a single vector injection (*$p < 0.05$).

Anc80-MMUT + ImmTOR showed more substantial weight gain over time following the second dose compared to mice treated with Anc80-MMUT alone (Figure 3). The effect of repeat dosing of Anc80-MMUT + ImmTOR was even more pronounced when biochemical markers were analyzed (Figure 4). As expected, repeat administration of Anc80-MMUT alone had no additional benefit on pMMA levels. In contrast, both groups treated with Anc80-MMUT combined with ImmTOR showed further, but differential, reduction of pMMA. This effect was dose-dependent: pMMA concentration in the group treated with low-dose ImmTOR decreased by a further 20%–40% and persisted at this level for 2 months, while pMMA concentration in the group co-treated with high-dose ImmTOR decreased by a further 30%–50% and persisted at this level for the duration of the study (~300 days). Pre-boost pMMA levels at day 55 in groups treated with Anc80 alone or combined with low-dose ImmTOR were not statistically different from post-boost levels due to high variability in baseline pMMA levels (Figure 4A); however, there was a clear trend toward lower pMMA levels after re-dosing in the low-dose ImmTOR-treated group (234 ± 96 μM at day 55 versus 171 ± 58 μM at day 70) compared to the vector alone group (345 ± 271 μM at day 55 versus 378 ± 257 μM at day 70). In contrast, the levels of pMMA in the high-dose ImmTOR group were significantly lower at day 70 after re-dosing (147 ± 34 μM) compared to day 55 (264 ± 100 μM). The relative levels of pMMA in the high-dose ImmTOR group were significantly different from those in the group treated with Anc80-MMUT alone at every time point measured after re-dosing (Figure 4B). Cumulatively, the effect of two doses of Anc80-MMUT + high-dose ImmTOR resulted in a 91% reduction of pMMA from baseline compared to a 79% reduction for the group treated with Anc80-MMUT alone, with the overall therapeutic benefit of the second dose on pMMA being inversely correlated with anti-Anc80 IgG antibody levels present in animals prior to re-dosing (Figure 1D).

FGF21 levels increased more than 2-fold after the repeat administration of Anc80-MMUT alone, and this increase persisted for the duration of the study (Figure 4C). In contrast, both groups of MMUT-MCK mice treated with a combination of Anc80-MMUT and ImmTOR showed low FGF21 levels that were maintained or further decreased after the second dose. Cumulatively, the effect of two doses of Anc80-MMUT + high-dose ImmTOR resulted in an 85%–93% reduction of FGF21 from baseline compared to a 70% reduction for the group treated with Anc80-MMUT alone.

Anc80-MMUT liver cell transduction efficiency was analyzed at the termination of the study, approximately 250 days after the second treatment (Figure 4D). Again, higher levels of vg/cell were detected in mice treated with a combination of Anc80-MMUT and ImmTOR. Specifically, there was a trend in favor of the higher dose of ImmTOR after a long-term follow-up of re-treated animals (Figure 4D). Transcription of *MMUT* mRNA in the liver after re-dosing was assessed separately and was also higher in mice treated twice with Anc80-MMUT + ImmTOR compared to animals treated with vector alone (Figure S4).

The inverse correlation of pMMA and FGF21 levels versus AAV transduction and transcription efficacy was further confirmed in a separate long-term study, in which two groups of mice were again repeatedly treated with 2.5E12 vg/kg Anc80-MMUT alone or with a high dose of ImmTOR (Figures 5A–5D). In this study, both groups were also administered dexamethasone at the time of vector administration since it was reported to inhibit AAV antibody formation and improve AAV pharmacokinetics.[34,35] Overall, dexamethasone did not appear to have a substantial effect on AAV therapeutic benefit with day 14–87 pMMA levels with or without ImmTOR and was similar to effects seen in the previous study (compare Figures 1B and 4A versus Figure 5A; a summary of day 14 data is shown in Table S1). As before, both treatment groups exhibited initial strong therapeutic benefit as evidenced by decreases in pMMA and FGF21 levels, while the benefit on pMMA levels was more pronounced in mice treated with Anc80-MMUT + ImmTOR (Figure 5A). Plasma FGF21 levels were also generally lower in animals treated with Anc80-MMUT + ImmTOR compared to those treated with vector alone, particularly after re-dosing on day 55 (Figure 5B). The spike in FGF21 on day 87 was due to an animal death in the vector alone group (the only MCK-MMUT-treated mouse lost after treatment in all studies reported herein).

Levels of viral DNA and M*MUT* mRNA assayed at 1 year after the study initiation were markedly higher and statistically different in mice treated with two doses of Anc80-MMUT + ImmTOR compared to mice treated with vector alone (Figures 5C and 5D). There was an

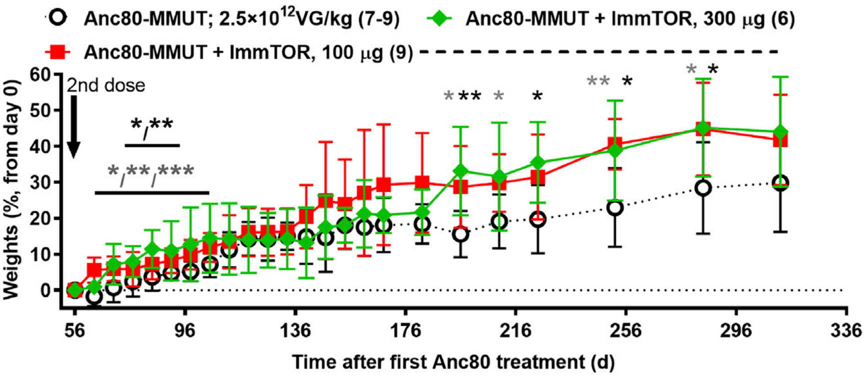

Figure 3. Weight gains in *Mmut*$^{-/-}$;Tg$^{INS-MCK-Mmut}$ mice after repeat treatment with Anc80-MMUT at 2.5 × 10^{12} vg/kg combined with ImmTOR

Gains (in % increase versus pre-re-dosing weight) in mice co-treated either with 100 or 300 μg of ImmTOR are shown versus those in mice treated only with Anc80-MMUT. The number of mice in each group is shown in parentheses (two mice treated with Anc80-MMUT alone were taken from the study into breeding 30 days after re-dosing). Time points with statistically significant (*p < 0.05, **p < 0.01, ***p < 0.001) weight differences versus group not receiving ImmTOR are indicated (gray and black symbols stand for 100 and 300 μg of ImmTOR, respectively). Dashed line indicates the interval during which collective weights in mice co-treated with ImmTOR (100 and 300 μg) show statistical difference (p < 0.01–0.05) versus those in mice treated only with Anc80-MMUT. Averages with SD are shown.

inverse correlation between viral DNA copy number and long-term pMMA levels, which was especially pronounced when data for both long-term studies were combined (Figure 5E). A similar correlation was seen in the subset of animals sacrificed at day 30 after a single dose (Figure S5A). Notably, this inverse correlation of long-term pMMA and viral DNA copy number was statistically significant even when data for both long-term studies were analyzed separately (Figures S5B and S5C).

Long-term histopathology after repeat treatments of MCK-MMUT mice with Anc80-MMUT alone or combined with ImmTOR

Findings in livers from MCK-MMUT mice were consistent with those reported previously by some of us (I.M., R.J.C., and C.P.V.) and included inflammatory cell infiltration, as well as vacuolization and intracytoplasmic inclusions within hepatocytes.[9,36] Cytoplasmic inclusions were round and single or multiple and composed of homogeneous, hyalinized pale to bright pink material within hepatocyte cytoplasm, which is most consistent with megamitochondria.[9,36] Additional findings included perivascular mononuclear cell infiltration and mixed cell inflammation; these findings were seen in both homozygous (data not shown) and heterozygous control mice, which do not exhibit gross pathology (Figure 6A; Figure S6A).

All treatment groups showed a substantial reduction in histopathological features of MMA. However, there were clear differences between treatment arms. Specifically, hepatocyte vacuolization and cytoplasmic inclusions were the most severe in MCK-MMUT mice receiving repeated administrations of Anc80-MMUT alone or with dexamethasone compared to groups dosed with ImmTOR (compare Figures 6B and 6E versus Figures 6C, 6D, and 6F, and Figures S6B and S6E versus Figures S6C, S6D, and S6F). Mononuclear cell infiltration as well as random infiltration of mixed inflammatory cells and hepatocytes with intracytoplasmic inclusions were consistently observed in these groups, although at lower levels than those observed in untreated MCK-MMUT mice, indicating therapeutic activity of Anc80-MMUT (Figure 6G). Collectively, mean pathology scores in liver were lowered in animals treated with Anc80-MMUT + ImmTOR versus vector alone, especially with respect to cytoplasmic inclusions and perivascular inflammatory cell infiltrate (Figure 6G and also Figure S6D versus heterozygous mouse shown in Figure S6A). A single mock-treated mouse surviving to 1 year exhibited more severe findings in both the liver and kidney, which included hepatocyte and renal tubular epithelial inclusions and degenerative lesions. This animal had also severe hepatocellular vacuolization and intracytoplasmic inclusions (scores of 2 and 4, correspondingly; Figure 6G), although without infiltrates, and it was the only one in the study with single-cell necrosis/apoptosis. No gross pathological differences were seen in kidneys or spleens of all treated animals, while robust tubular dilation, vacuoles, cytoplasmic inclusions, and tubular degeneration/necrosis were seen in the kidney from a single non-treated mouse.

Anti-Anc80 IgG dynamics

The dynamics of anti-AAV IgG development was followed up for the duration of both long-term studies using the same Anc80-MMUT dose of 2.5 × 10^{12} vg/kg (Figures 1, 2, 3, 4, and 5). There was a marked additional elevation of AAV IgG titers after the second vector administration without ImmTOR, while the IgG response in groups treated with ImmTOR stayed manifestly depressed and still was not detectable up to 2 months after repeat dosing (Figure 7). Importantly, antibody titers remained low even more than 250 days after the second administration. In both ImmTOR-treated groups IgG levels were statistically different from those in the group receiving Anc80-MMUT alone at all time points tested. Similar results were obtained combining ImmTOR with a higher vector dose of 5.0 × 10^{12} vg/kg (Figure S7). Contrary to the earlier reports,[34] dexamethasone alone was ineffective in inhibiting anti-AAV antibody formation (Figure S8). The combination of ImmTOR + dexamethasone inhibited antibody formation through day 87; however, by day 126 mice developed anti-Anc80 IgG antibodies.

DISCUSSION

Gene therapy is rapidly becoming a clinically relevant option for the treatment of inborn errors of metabolism, especially those with

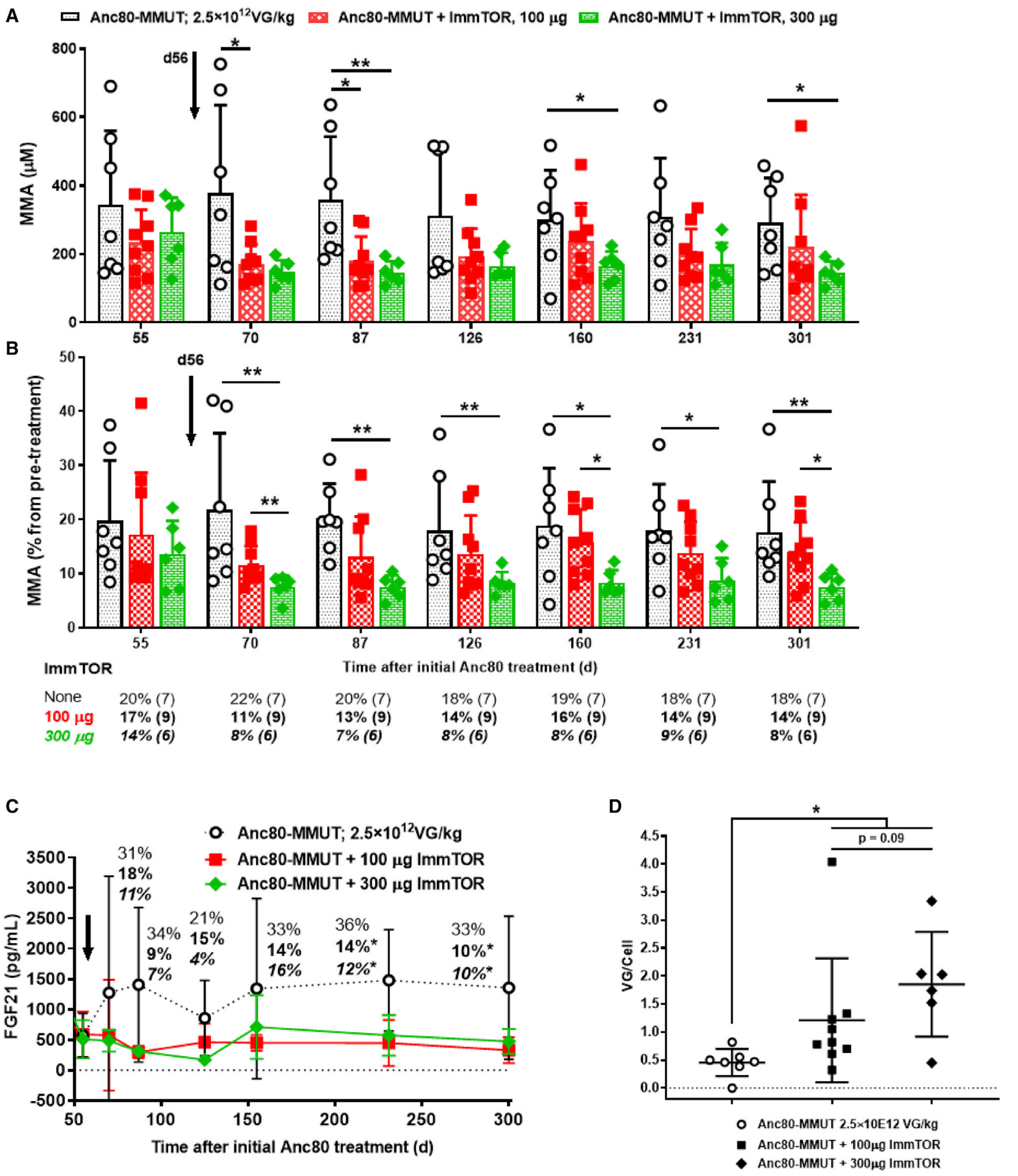

Figure 4. Therapeutic benefit of repeat dosing with Anc80-MMUT combined with ImmTOR in *Mmut*$^{-/-}$;Tg$^{INS\text{-}MCK\text{-}Mmut}$ mice

(A and B) Dynamics of absolute (A) (in μM) and relative (B) (versus those prior to initial day 0 treatment as 100%) plasma levels of methylmalonic acid before and after therapeutic Anc80-MMUT re-dosing on day 56 (indicated by arrow) with statistical significance indicated (*$p < 0.05$, **$p < 0.01$). The number of mice per each group at each time point is shown in parentheses. (C) Dynamics of plasma FGF21 levels after repeat Anc80-MMUT injection (day 56, indicated by an arrow). Relative levels (versus

(legend continued on next page)

hepatic involvement. Most, if not all, of these disorders, such as IMMA, require immediate management and early intervention to prevent subsequent tissue and organ damage. This exact approach has been recently proven to dramatically change the outcomes of otherwise uniformly fatal neurodegenerative conditions, such as spinal muscular atrophy (SMA) type 1.[37] Moreover, it is likely that any patient treated as an infant will require additional therapeutic interventions during their lifetime due to the natural growth of target tissue or organ. Notably, AAV-based vectors, which have been recently approved by the US Food and Drug Administration (FDA) for the treatment of several genetic disorders[38,39] are very immunogenic and require high systemic doses for effects, which further emphasizes the need to consider immune modulation as a critical component of systemic AAV gene therapy. Specifically, it is certain that repeat systemic gene therapy in pediatric patients using AAV vectors will not be effective (and may potentially even be detrimental) due to *de novo* formation of vector-induced Nabs against capsid proteins, possibly accompanied by induction of other adaptive immune responses to vector and transgene.[31,40] Therefore, the ability to mitigate the immune response to AAV vectors in a targeted fashion is a highly desirable tool for a future gene therapeutic armamentarium and may potentially cover a broad range of applications.

ImmTOR particles encapsulating rapamycin were designed to mitigate the formation of anti-drug antibodies against co-administered biologic drugs through the induction of tolerogenic dendritic cells and antigen-specific regulatory T cells (Tregs).[41,42] This approach has been validated with multiple biologic therapies in many models and preclinical settings,[41–45] and it is currently being investigated in a phase 3 clinical trial in combination with a recombinant uricase enzyme for the treatment of chronic refractory gout. The application of ImmTOR to gene therapy is designed to address one of the key limitations of systemic AAV gene therapy, namely, the inability to re-treat patients due to the formation of Nabs.[28,40,46] The inability to re-treat is of particular concern for liver-directed gene therapy of metabolic diseases, as the liver is a regenerative organ and hepatocyte proliferation related to growth in pediatric patients or in response to injury or infection could result in the loss of transgene expression. At the same time, severely afflicted patients need to be treated early in life to avoid irreversible pathologic consequences.[31]

In the current study, we demonstrate the benefit of combining ImmTOR with AAV gene therapy in a mouse model of IMMA. While a therapeutic dose of Anc80-MMUT showed long-term efficacy in the MCK-MMUT model, similar to that previously reported, the inclusion of ImmTOR provided additional therapeutic benefit both at the first dose and with repeat dosing. Recent studies have indicated that the benefit of ImmTOR on first dose transgene expression is mediated through a mechanism that is independent of the enabling benefit of ImmTOR on re-dosing.[29] The first dose benefit of ImmTOR appears to be mediated by enhanced AAV liver trafficking and is dependent on admixing ImmTOR + AAV prior to dosing. In addition, induction of autophagy by rapamycin in hepatocytes has also been shown to enhance viral transduction.[30] In the MMUT-MCK model of IMMA, the addition of ImmTOR to Anc80-MMUT vector results in a dose-dependent maintenance of low serum MMA levels at day 30 (Figure 1B) and increased vector genome copies per cell in the liver (Figure 2A). Importantly, the addition of ImmTOR to Anc80-MMUT also inhibits the formation of anti-Anc80 IgG antibodies (Figure 1D; Figure S8), which enabled productive re-dosing of the gene therapy vector, indicating the suppression of Nabs. A limitation of this study is that anti-IgM antibodies and *in vitro* neutralizing activity were not assessed due to the limited blood volume obtainable from juvenile MMUT-MCK mice. However, previous reports indicate a high degree of correlation between anti-AAV IgG levels and Nab titers.[47,48] Moreover, Nab titers are a surrogate for *in vivo* neutralizing activity, and in our studies, we demonstrated *in vivo* neutralizing activity by assessing the ability of ImmTOR to enable vector re-dosing, as determined by increased vector genome copy numbers in the liver, increased hepatic mRNA expression, increased weight gain, and reduced plasma methylmalonic acid and FGF21 levels following vector re-administration. Mice treated with ImmTOR + dexamethasone developed anti-AAV IgG antibodies by day 126. Further exploration of immunomodulating regimens that either adversely affect or prolong the benefit of ImmTOR is warranted. Re-dosing of Anc80-MMUT + ImmTOR results in sustained reduction of plasma MMA and FGF21, increased hepatic *MMUT* mRNA expression, further increases in hepatic vector genome copies, and alleviation of hepatic pathology (Figures 3, 4, 5, and 6). The benefit of ImmTOR at re-administration is dose-dependent, with 300 μg being superior to 100 μg despite similar levels of inhibition of anti-Anc80 IgG antibodies. The reason for the enhanced benefit at the higher ImmTOR dose is not clear, but it may be related to increased induction of hepatic autophagy by rapamycin which, in turn, has been shown to increase AAV transduction.[29,30]

The challenge for re-dosing AAV vectors in humans is that the levels of Nabs that develop in response to initial gene therapy administration are typically much higher than those that occur after natural exposure to wild-type AAV virus. Moreover, long-term follow-up of patients treated with AAV2 gene therapy for hemophilia B showed a persistent high titer of Nabs sustained for up to 15 years.[49] Since even low antibody titers can inhibit AAV transduction,[40] enabling vector re-dosing requires nearly complete inhibition of antibody

pre-treatment day 0 levels as 100%) are also shown for each group at every time point with statistical significance indicated (*p < 0.05). Averages with SD are shown in all graphs. Data for mice treated with Anc80-MMUT alone are shown in normal font, those treated with Anc80-MMUT combined with 100 μg of ImmTOR are in bold, and those treated with Anc80-MMUT combined with 300 μg of ImmTOR are in bold and italicized. (D) Long-term AAV-driven transduction of liver cells of $Mmut^{-/-}$;$Tg^{INS-MCK-Mmut}$ mice after repeat dosing with Anc80-MMUT combined with ImmTOR (100 or 300 μg) measured as vg/cell at approximately 250 days after therapeutic re-dosing (300–310 days after initial treatment) with Anc80-MMUT either alone or combined with 100 or 300 μg of ImmTOR. Statistical significance (*p < 0.05) is shown with p values between selected groups also indicated. Averages with SD are shown in all graphs.

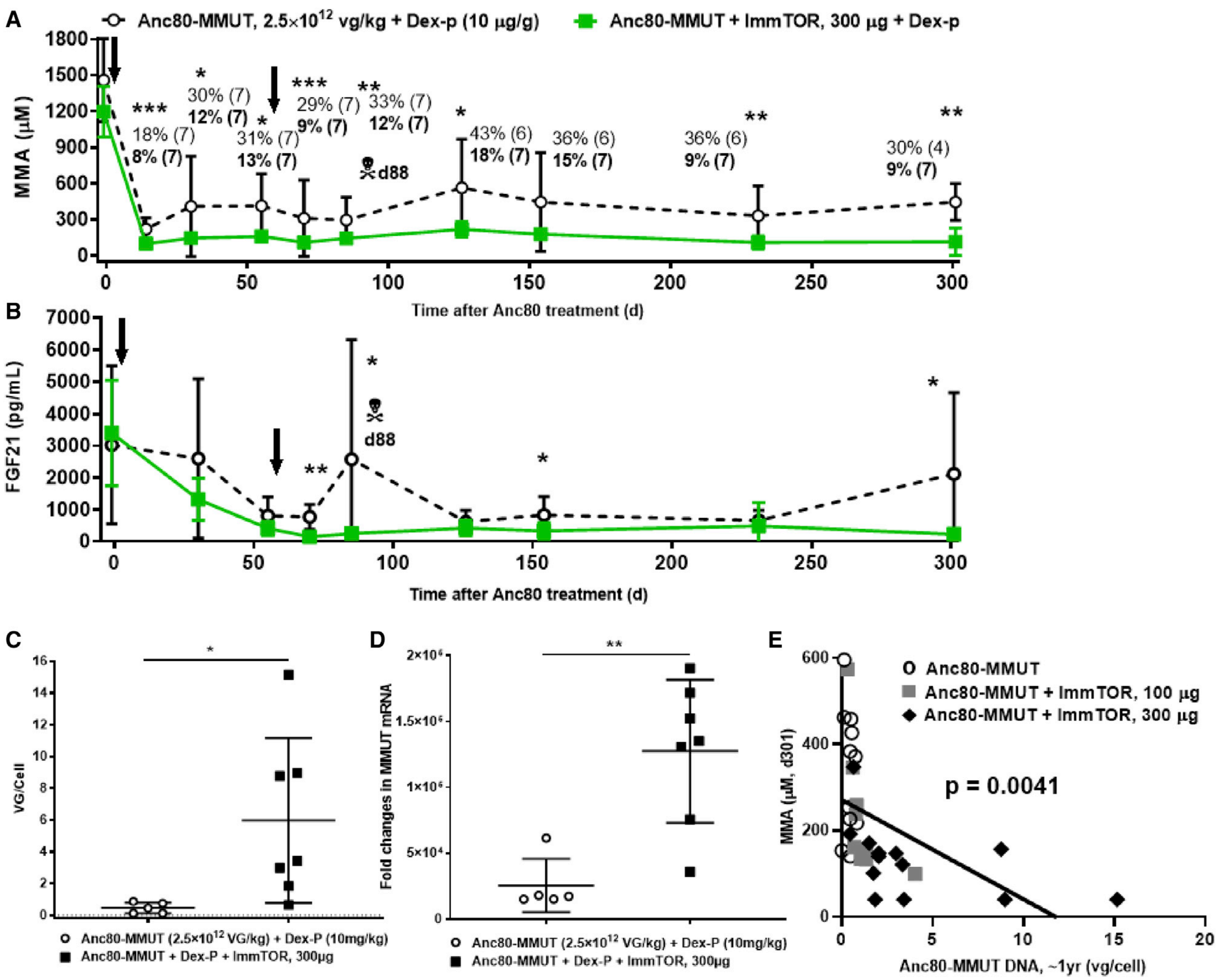

Figure 5. Therapeutic effect of repeat Anc80-MMUT dosing correlates with long-term liver cell transduction and *MMUT* mRNA expression

Two groups of mice were treated twice (days 0 and 56, indicated by arrows) with Anc80-MMUT alone or combined with 300 μg of ImmTOR and followed up for 300 days. A single fatality (shown in A and B) occurred in the group treated with Anc80-MMUT alone on day 88 after initial inoculation. Both groups received 10 mg/mL dexamethasone concurrently with Anc80 inoculation. (A) Dynamics of pMMA levels. Relative pMMA levels (versus pre-treatment levels as 100%) are also shown for each group at every time point. Time points with statistically significant (*$p < 0.05$, **$p < 0.01$, ***$p < 0.001$) differences between the groups indicated. Number of mice per group at each time point is shown in parentheses. (B) Dynamics of plasma FGF21 levels. Time points with statistically significant (*$p < 0.05$, **$p < 0.01$) differences between the groups are indicated. (C) Efficacy of Anc80-MMUT transduction of $Mmut^{-/-}$;$Tg^{INS\text{-}MCK\text{-}Mmut}$ mouse livers (in vg/cell) at approximately 300 days after therapeutic re-dosing with Anc80-MMUT either alone or combined with 300 μg of ImmTOR (340–360 days after initial treatment). Statistical significance (*$p < 0.05$) is shown. (D) Efficacy of *MMUT* mRNA transcription in $Mmut^{-/-}$;$Tg^{INS\text{-}MCK\text{-}Mmut}$ mouse livers (as measured by fold increase over the baseline, GAPDH normalized) at the same time point as in (C). Statistical significance (**$p < 0.01$) is shown. Averages with SD are shown in all graphs. (E) Inverse correlation of AAV long-term transduction efficacy and pMMA levels. Summary of two individual studies conducted using similar protocols and identical Anc80 doses and injection schedule (with or without dexamethasone). Data presented in Figures 4A, 4C, 5A, and 5C are used for analysis with no samples omitted. The p value of correlation is shown.

formation. Various strategies have been described to enable re-dosing or dosing of intravenously administered AAV in the presence of pre-existing antibodies, such as combination immunosuppressive drug therapy,[31,50–52] decoy empty capsids[53], plasmapheresis,[54–58] and most recently an IgG-specific protease.[59] Some immunosuppressive regimens are inadequate to enable re-dosing, and some specific immunosuppressive drugs may be counterproductive by suppressing Tregs as well as effector T cells.[60] Others appear effective in preclinical studies but require chronic immunosuppression with multiple agents. The use of empty decoy capsids is limited by the cost of production and has the potential to exacerbate cellular immune responses associated with liver inflammation and loss of transgene expression. Plasmapheresis[55–58] and IgG protease[59] appear to be effective for transiently depleting total IgG antibodies in circulation in preclinical

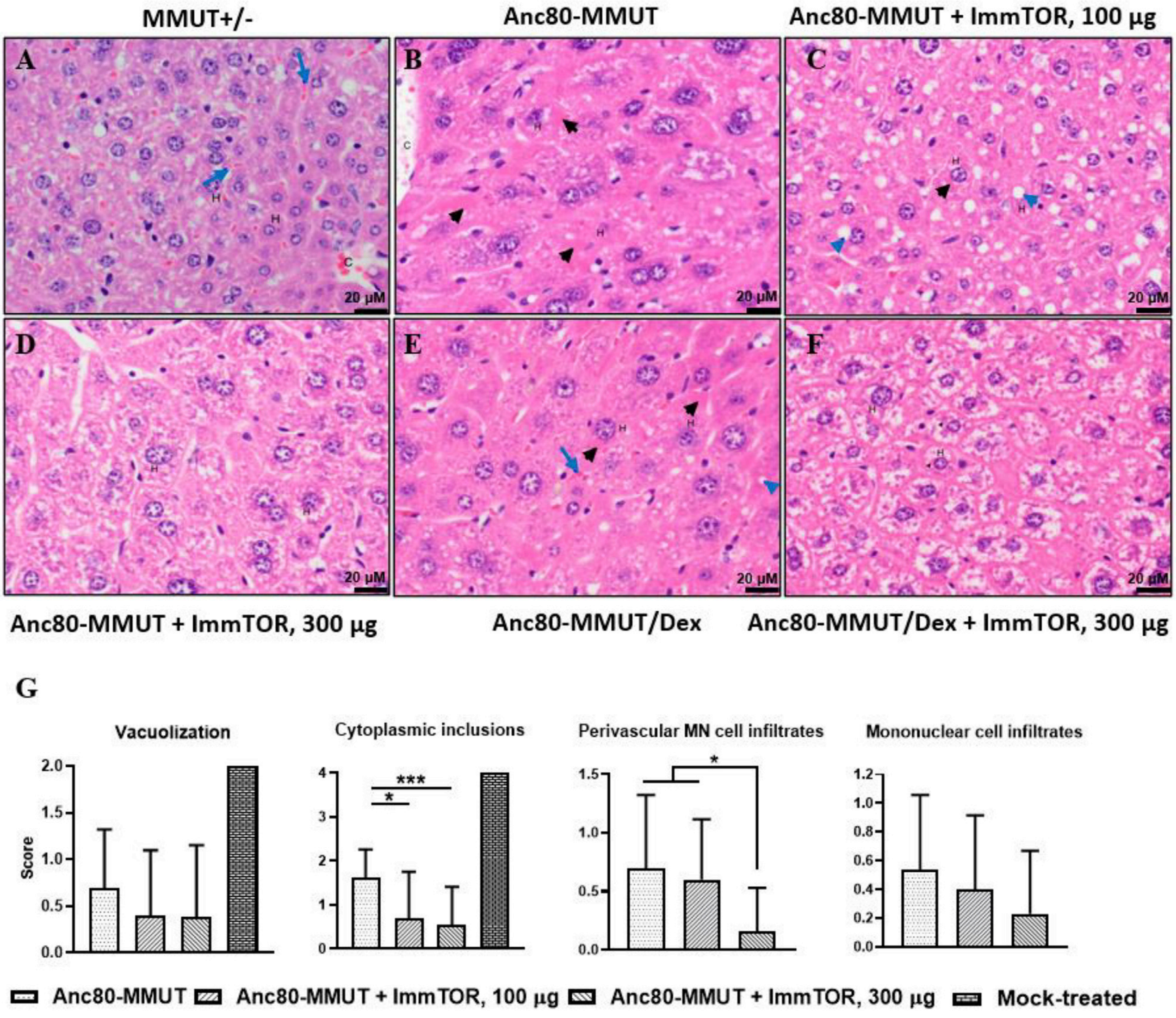

Figure 6. Long-term alleviation of hepatic pathology by Anc80-MMUT and ImmTOR

Representative photomicrographs of liver sections from heterozygous $MMUT^{+/-}$ (A) and Anc80-MMUT-treated $Mmut^{-/-}$;$Tg^{INS-MCK-Mmut}$ mice (B–F) are shown (original magnification, ×400). Anc80-MMUT (days 0 and 56) was used alone (B) or combined with 100 μg (C) or 300 μg (D) of ImmTOR, combined with dexamethasone (E) or dexamethasone and 300 μg of ImmTOR (F). Hematoxylin and eosin staining of liver sections from 1-year-old mice are shown. Scale bars, 20 μm. In heterozygous control (A), hepatocytes (H) lack vacuoles and intracytoplasmic inclusions; pale-staining cytoplasm shows the presence of glycogen, which is within normal limits. Sinusoids containing erythrocytes (blue arrows) between hepatocyte cords and a small central vein (C) are indicated. In mice treated with Anc80-MMUT without ImmTOR (B and E), numerous hepatocytes have hyalinized with moderately intense eosinophilic pink inclusions (black arrowheads) within their cytoplasm or small round non-staining vacuoles (blue arrowhead). A sinusoid containing erythrocytes (blue arrow) is indicated in (E). Mice co-treated with Anc80-MMUT combined with low dose ImmTOR (C) possess hepatocytes that contain only rare eosinophilic inclusions and more numerous non-stained vacuoles, while in those co-treated with high-dose ImmTOR (D and F) histologic findings are minimal to absent. (G) Mean histopathology scores with statistical significance shown (*$p < 0.05$, ***$p < 0.001$); group size is 10–13 mice (except for mock-treated control, 1 mouse); mice receiving the same amounts of ImmTOR (0, 100, or 300 μg) in both studies are unified in a single group.

studies. However, a small clinical study showed that even five rounds of plasmapheresis was only effective in reducing Nabs to background levels with low-moderate titers of pre-existing Nabs.[54] Similarly, the IgG protease was effective with low levels of pre-existing antibodies (titers 3–17), but appeared less effective in a setting of re-dosing, where pre-existing Nab titers >1,000 were only reduced to titers >100.[59] Moreover, neither plasmapheresis nor IgG protease affects the underlying T cell response to the capsid. Similarly, daily administration of free rapamycin for more than 3 weeks was only partially efficient in prevention of immune response to AAV vector in a rat model of Crigler-Najjar syndrome and was completely ineffective in animals with prior AAV exposure.[31]

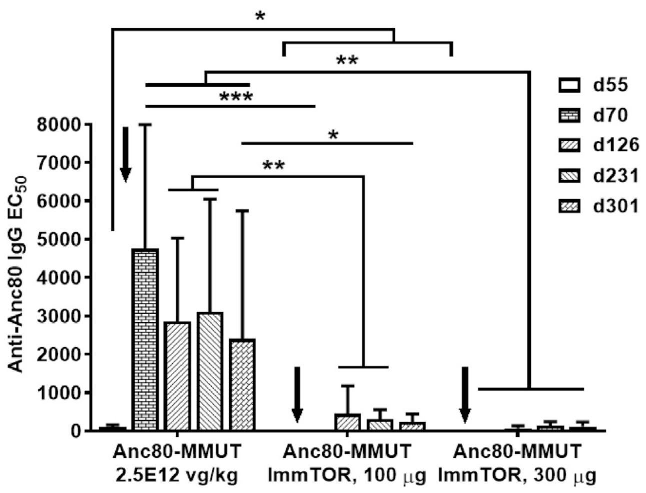

Figure 7. ImmTOR suppresses the induction of IgG against Anc80 vector after initial and repeat co-administrations

Groups of mice (six to nine per group at all of the following time points) were injected twice with an 8-week interval with 2.5×10^{12} vg/kg of Anc80-MMUT alone or combined with ImmTOR (at 100 or 300 μg of rapamycin; see Figures 1, 2, 3, and 4) and Anc80 IgG in serum measured at times indicated and presented as EC_{50}. Averages with SD are shown. Statistical difference between the group treated with Anc80-MUT alone versus those treated with Anc80 combined with ImmTOR is shown for all time points (*p < 0.05, **p < 0.01, ***p < 0.001).

In contrast, ImmTOR has been shown to inhibit both the T and B cell response to AAV capsid.[28,29] The repeated co-administration of Anc80 and ImmTOR was well tolerated and continued to completely inhibit IgG antibodies to AAV vector for more than 2 months after re-dosing with minimal antibody elevation seen by the study completion nearly a year after initial intervention (Figure 7; Figure S7). Moreover, we have shown that ImmTOR particles enable productive transduction in the presence of low levels of AAV Nabs *in vivo*, most likely by shielding the virions and enhanced liver targeting.[29] ImmTOR has been dosed in more than 250 patients and been shown to be effective in inhibiting anti-drug antibodies against an immunogenic recombinant uricase enzyme of fungal origin. However, the ability of ImmTOR to inhibit anti-AAV Nab formation in humans remains to be evaluated.

The systemic AAV gene therapy for IMMA is a compelling application of ImmTOR, as severely affected IMMA patients must be treated early in childhood to stabilize the metabolic defect and minimize disease progression. Thus, IMMA patients are likely to require dosing later in life. The addition of ImmTOR to AAV gene therapy for IMMA also provides a first dose benefit, which is manifested by sustained and more complete reduction of plasma MMA and FGF21. Recent studies indicate that admixing ImmTOR with AAV prior to their administration increases hepatocyte transduction.[29] Moreover, induction of autophagy in the liver by rapamycin may be beneficial in transiently attenuating metabolic instability (P.O.I., unpublished data), possibly by improving defective mitophagy observed in IMMA patients and IMMA mouse models.[61]

This multi-pronged mechanism of ImmTOR action makes it an attractive candidate to enhance systemic gene therapeutic applications, particularly in those clinical indications where repeat vector dosing may be necessary. Liver-directed gene therapy for metabolic diseases in pediatric patients, from infancy to early adolescence, presents a strong case for ImmTOR co-administration, since on one hand early intervention is highly desirable to prevent disease progression, but on the other hand the target organ is destined to substantially grow over time, resulting in vector dilution.

The benefit of adding ImmTOR to AAV gene therapy is both immediate and long-term, dose-dependent, and not capsid-specific.[29] It is characterized by increased vector copy number in liver cells and elevated synthesis of transgene-encoded mRNA. The rapid and enhanced transgene expression may enable therapeutic benefit at lower doses of AAV and faster onset of the therapeutic effects. Importantly, mitigating the formation of anti-AAV IgG antibodies by ImmTOR provides an ability to re-dose vector over time at a different dose levels in order to maintain or restore the therapeutic efficacy of gene therapy.

MATERIALS AND METHODS

Study design

The objective was to investigate the effects of ImmTOR (nano-encapsulated rapamycin) on AAV vector transduction *in vivo* after initial and repeat dosing. Research subjects were animals and their tissues. Sample size in animal experiments was determined before the study as per Institutional Animal Care and Use Committee (IACUC) guidelines. No outliers were excluded except for a single individual mouse, which was of the smallest size at the time of administration and was documented not to receive a full dose of Anc80-MMUT. All experiments were controlled animal studies or controlled laboratory experiments. Due to the limited availability of $Mmut^{-/-}$;$Tg^{INS\text{-}MCK\text{-}Mmut}$ mice, subjects in each individual study were taken into an experiment at different dates and each experimental group (to which animals were randomly distributed) contained both males and females. Both short-term and long-term studies with therapeutic viral vector administration were repeated twice with minimal variations.

Mice

The colony of $Mmut^{-/-}$;$Tg^{INS\text{-}MCK\text{-}Mmut}$ mice was established with breeders received from the National Institutes of Health, National Human Genome Research Institute (NHGRI, Bethesda, MD, USA).[9] These mice are designed to express MMUT in a muscle-specific fashion to rescue the lethal phenotype displayed by $Mmut^{-/-}$ knockout mice, but at the same time to allow disease manifestations in the tissues that lack the enzyme. These mice have been originally generated using the MCK promoter to drive the expression of a *MMUT* cassette. Mice were bred in-house using homozygous $Mmut^{-/-}$;$Tg^{INS\text{-}MCK\text{-}Mmut}$ males and heterozygous $Mmut^{+/-}$;$Tg^{INS\text{-}MCK\text{-}Mmut}$ females since homozygous knockout females are generally non-productive. Breeding was followed by offspring genotyping (days 21–25) to identify $Mmut^{-/-}Tg^{INS\text{-}MCK\text{-}Mmut}$ animals, which are viable but display massive elevations of serum metabolites and severe growth retardation leading to ~60%–70% short-term survival unless treated. Immunologically

naive male or female $Mmut^{-/-}$;$Tg^{INS\text{-}MCK\text{-}Mmut}$ mice aged 24–28 days (or weight ranging from 6 to 14 g for females and from 7 to 15 g for males) were taken into studies with the exception of a single study of an early treatment scheme with a subtherapeutic dose Anc80-MMUT, in which 14-day-old mice (weight ranging from 7 to 12 g for females and males) were used. Mice were weighed 24 h prior to vector administration, and the Anc80-MMUT dose was calculated individually based on these measurements. All of the experiments were conducted in strict compliance with the NIH *Guide for the Care and Use of Laboratory Animals* and other federal, state, and local regulations and were approved by Selecta's IACUC.

Mouse genotyping

At the time of weaning (21 days), mice were ear punched creating a 3-mm disk of tissue. The tissue was then placed in a 96-well plate and sent overnight to Transnetyx (Cordova, TN, USA) for genotyping. Transnetyx analyzed three probes to determine the genotype of the mice, the results of which were available within 72 h. PCR amplifications were performed across the loxP site of the targeted allele, as well as across the *Mmut* cDNA to detect the INS-MCK-*Mmut* transgene. Primers used were as follows: 5′-loxP site, 5′-CCATTCTGGGA AGGCTTCTA-3′; 3′-loxP site, 5′-TGCACAGAGTGCTAGTT TCCA-3′. Detection of the INS-MCK-*Mmut* transgene was completed by amplification across the *Mmut* cDNA with the following primers: forward, 5′-CATGTTGAGAGCTAAGAATC-3′; reverse, 5′-TAGAAGTTCATTCCAATCCC-3′.

Viral vectors

Anc80-hAAT-MMUT was manufactured by SAB Tech (Philadelphia, PA, USA) using proprietary helper plasmid, Anc80AAP plasmid, and the plasmid containing the wild-type human *MMUT* gene (used in all experiments unless otherwise noted) or its modified version *syn-MMUT*[62] under the control of liver-specific hAAT (human alpha-1 antitrypsin) promoter. The plasmids were transfected into adherent HEK293 cells and harvested at 72 h post-transfection by cell lysis. The clarified supernatant from the harvest was purified by $CsCl_2$ gradient, and the fraction containing full Anc80 vector particles was collected. The viral vector band was assayed by SDS-PAGE gel and silver stain to determine a viral titer. Standard vector dose used in all studies unless otherwise described was 2.5×10^{12} vg/kg.

ImmTOR particles

ImmTOR polylactic acid (PLA)-based polymer nanoparticles encapsulating rapamycin were manufactured as described earlier[41,42] and administered in doses of 100 or 300 μg of rapamycin per mouse. Rapamycin (sirolimus) was manufactured by Concord Biotech (Ahmedabad, India).

Animal injections and tissue collection

Mice were injected (intravenously [i.v.], via retro-orbital plexus) either with Anc80-MMUT alone or combined as an admixture with ImmTOR at 100 or 300 μg of rapamycin per mouse. In the latter case, ImmTOR at the desired concentration was equilibrated to room temperature, and then viral vector was added to it and the resulting combination of vector/ImmTOR was mixed thoroughly (inverted at least three times and then three times more immediately before injection) and administered within 10–30 min. Mice were bled via retro-orbital route into EDTA-coated plasma tubes, samples were spun at 5,000 rpm for 10 min, and then 5 μL of plasma was added into 195 μL of H_2O (1:40) for pMMA analysis and the rest of each sample was placed into a separate tube for in-house FGF21 or IgG analysis and stored at −20°C. At study termination, livers and kidneys of experimental animals were collected and processed for histological analysis with liver tissues being also processed separately to assay vector transduction level and *MMUT* mRNA as described below. For histological tissue collection, liver sections and kidneys were placed into cassettes in 10% neutral buffered formalin (NBF) and shipped to HistoTox Labs (Boulder, CO, USA) for data analysis. For qPCR biodistribution and qRT-PCR tissue collection, tissue sections were placed into 1 mL of RNALater and stored at −20°C prior to in-house analysis.

Histological analysis

Liver, spleen, and kidney samples from 36 $Mmut^{-/-}$;$Tg^{INS\text{-}MCK\text{-}Mmut}$ mice treated with Anc80-MMUT alone or combined with ImmTOR and 2 heterozygous $Mmut^{+/-}$ mice, all around 1 year old, were fixed and paraffin embedded separately to create 91 total blocks. One slide per block was sectioned and stained with hematoxylin and eosin. Glass slides were evaluated by a board-certified veterinary pathologist in a non-blinded fashion, using light microscopy. Histologic lesions in each tissue segment were graded for severity (0–5) as follows: 0, absent; 1, minimal; 2, mild; 3, moderate; 4, marked; 5, severe.

Methylmalonic acid and FGF21 concentrations in plasma

Plasma samples (1:40 dilution) were frozen and sand analyzed for pMMA levels by mass spectrometry at Sannova Analytical (Somerset, NJ, USA). FGF21 concentration was determined by ELISA using the mouse/rat FGF21 commercial kit from R&D Systems (Minneapolis, MN, USA). Plasma samples were used at a 1:6 dilution.

IgG Anc80 ELISA

96-well plates were coated overnight with Anc80, washed and blocked on the following day, followed by sample incubation (1:40 diluted serum added to the plate). Plates were then washed, and the presence of IgG was detected using rabbit anti-mouse IgG-specific horseradish peroxidase (HRP) (Jackson ImmunoResearch, West Grove, PA, USA). The presence of rabbit anti-mouse IgG-specific HRP was visualized using tetramethylbenzidine (TMB) substrate and measuring an absorbance at 450 nm with a reference wavelength of 570 nm. The optical density (OD) observed is reported and is proportional to the quantity of anti-Anc80 IgG antibody in the sample. The half-maximal effective concentrations (EC_{50}s) were determined as follows. The positive control antibody (Fitzgerald anti-AAV8-IgG) and samples were diluted 1:40 followed by a 1:3 serial dilution and the assay was performed as described above. The EC_{50} was calculated using the four-parameter logistic curve fit function in the SoftMax Pro software program. The positive control antibody (Fitzgerald anti-AAV8-IgG) was used as the standard curve to determine the EC_{50} for each sample.

qPCR liver biodistribution

Livers were harvested and tissue was stored in RNAlater at −20°C ± 5°C until the time of either vector genome biodistribution or mRNA expression analysis. qPCR biodistribution analysis was performed on the liver tissues by homogenization via BeadBug treatment, followed by DNA extraction using a QIAamp DNA mini kit according to the manufacturer's instructions. The extracted DNA was quantified using a Qubit fluorometer. 100 ng of extracted DNA per sample was used in each qPCR reaction. The primers and probe set used for qPCR were specific to the wild-type human *MMUT* transgene (forward primer sequence 5′-TACCTCTTATCTTCCTCCCACAGCTCC-3′, reverse primer sequence 5′-CCTGCCTCAGGTAATGAGGTGAAAGT-3′ and probe sequence 5′-ATTCTTAGCTCTTAACATGGTGGCG GC-3′). The standard curves used to interpolate the data were established using plasmid containing wild-type human *MMUT*.

qRT-PCR mRNA quantitation

For mRNA expression, qRT-PCR was used to compare the mRNA levels of wild-type *MMUT* or *synMU*T in livers from mice in groups treated with Anc80 carrying the hAAT promoter. RNA was extracted from liver tissues using QIAGEN's RNeasy kit, following the manufacturer's instructions. cDNA obtained from the extracted RNA was then used in a qPCR reaction to quantify the relative amount of wild-type *MMUT* or *synMUT* mRNA in a liver sample. For *MMUT* the following primers were used: forward, 5′-CAGTTGGAAA AAGAAGACGCTGTA-3′; reverse, 5′- ATCTGCCTGTTTCGC ACTGA-3′; and probe, 5′-TCTGGCAATTGATAATAC-3′. For *synMMUT* the primers were as follows: forward, 5′-TCAGTCCCTC CACACTAACT-3′; reverse, 5′- CCGCTTTCCTCCTGGATAAT-3′; and probe, 5′-CGCCCGGATAGCCAGAAATACTCAAA-3′. To normalize for differences in RNA extraction efficiencies between samples, the results were normalized to the amount of GAPDH mRNA in a liver sample.

Statistical analysis

Statistical analyses were performed using GraphPad Prism 8.0.2. To compare the mouse experimental groups pairwise, either a multiple t test (for several time points) or Mann-Whitney two-tailed test (for a single time point) was used. A one-way ordinary ANOVA was used to compare data from *in vitro* assays. Significance is shown in each figure legend (*$p < 0.05$, **$p < 0.01$, ***$p < 0.001$; not significant, $p > 0.05$). All data for individual experimental groups are presented as mean ± SD (error bars).

SUPPLEMENTAL INFORMATION

Supplemental information can be found online at https://doi.org/10.1016/j.omtm.2021.06.015.

ACKNOWLEDGMENTS

This work was supported by Selecta Biosciences. R.J.C., L.L., I.M., and C.P.V. were supported, in part, by the Intramural Research Program of the National Human Genome Research Institute in the National Institutes of Health (project 1ZIAHG200318-16 in which C.P.V. serves as the Principal Investigator). We thank Julie LePrevost for expert technical help and Fen-ni Fu for ImmTOR rapamycin content analysis.

AUTHOR CONTRIBUTIONS

P.O.I. and T.K.K. designed all experiments, analyzed and interpreted data, and wrote the manuscript; A.M.M., G.L.R., C.J.R., S.L.E., T.C., and A.C. conducted experiments; S.S.L. analyzed and interpreted data; and L.L., R.J.C., I.M., E.A.-M., L.P.M.J., L.H.V., and C.P.V. contributed reagents and edited the manuscript.

DECLARATION OF INTERESTS

P.O.I., A.M.M., G.L.R., C.J.R., S.S.L., S.L.E., T.C., A.C., L.P.M.J., and T.K.K. are employees and shareholders of Selecta Biosciences. E.A.-M is an employee and holds stocks of Akouos, Inc. L.H.V. received consulting fees and research funding from Selecta Biosciences and holds equity in and serves on the Scientific Advisory Board of Akouos. He is an inventor of Anc80L65, licensed to biopharmaceutical companies, including Selecta Biosciences, from which he receives royalties. C.P.V. received research funding from Selecta Biosciences. R.J.C., L.L., I.M., and C.P.V. are co-inventors on patents and patent applications filed by the NIH on their behalf.

REFERENCES

1. de Baulny, H.O., Benoist, J.F., Rigal, O., Touati, G., Rabier, D., and Saudubray, J.M. (2005). Methylmalonic and propionic acidaemias: Management and outcome. J. Inherit. Metab. Dis. *28*, 415–423.
2. Manoli, I., Sloan, J.L., and Venditti, C.P. (2005). Isolated methylmalonic acidemia. In GeneReviews, M.P. Adam, H.H. Ardinger, R.A. Pagon, S.E. Wallace, L.J.H. Bean, K. Stephens, and A. Amemiya, eds. (University of Washington, Seattle), pp. 1993–2020, [Internet].
3. Hörster, F., Baumgartner, M.R., Viardot, C., Suormala, T., Burgard, P., Fowler, B., Hoffmann, G.F., Garbade, S.F., Kölker, S., and Baumgartner, E.R. (2007). Long-term outcome in methylmalonic acidurias is influenced by the underlying defect (mut^0, mut^-, cblA, cblB). Pediatr. Res. *62*, 225–230.
4. Kölker, S., Valayannopoulos, V., Burlina, A.B., Sykut-Cegielska, J., Wijburg, F.A., Teles, E.L., Zeman, J., Dionisi-Vici, C., Barić, I., Karall, D., et al. (2015). The phenotypic spectrum of organic acidurias and urea cycle disorders. Part 2: The evolving clinical phenotype. J. Inherit. Metab. Dis. *38*, 1059–1074.
5. Chandler, R.J., and Venditti, C.P. (2019). Gene therapy for methylmalonic acidemia: Past, present, and future. Hum. Gene Ther. *30*, 1236–1244.
6. Matsui, S.M., Mahoney, M.J., and Rosenberg, L.E. (1983). The natural history of the inherited methylmalonic acidemias. N. Engl. J. Med. *308*, 857–861.
7. O'Shea, C.J., Sloan, J.L., Wiggs, E.A., Pao, M., Gropman, A., Baker, E.H., Manoli, I., Venditti, C.P., and Snow, J. (2012). Neurocognitive phenotype of isolated methylmalonic acidemia. Pediatrics *129*, e1541–e1551.
8. Kölker, S., Garcia-Cazorla, A., Valayannopoulos, V., Lund, A.M., Burlina, A.B., Sykut-Cegielska, J., Wijburg, F.A., Teles, E.L., Zeman, J., Dionisi-Vici, C., et al. (2015). The phenotypic spectrum of organic acidurias and urea cycle disorders. Part 1: The initial presentation. J. Inherit. Metab. Dis. *38*, 1041–1057.
9. Manoli, I., Sysol, J.R., Epping, M.W., Li, L., Wang, C., Sloan, J.L., Pass, A., Gagné, J., Ktena, Y.P., Li, L., et al. (2018). FGF21 underlies a hormetic response to metabolic stress in methylmalonic acidemia. JCI Insight *3*, e124351.
10. Oberholzer, V.G., Levin, B., Burgess, E.A., and Young, W.F. (1967). Methylmalonic aciduria. An inborn error of metabolism leading to chronic metabolic acidosis. Arch. Dis. Child. *42*, 492–504.
11. Stokke, O., Eldjarn, L., Norum, K.R., Steen-Johnsen, J., and Halovorsen, S. (1967). Methylmalonic acidemia: A newborn error of metabolism which may cause fatal acidosis in the neonatal period. Scand. J. Clin. Lab. Invest. *20*, 313–328.

12. Baumgartner, M.R., Hörster, F., Dionisi-Vici, C., Haliloglu, G., Karall, D., Chapman, K.A., Huemer, M., Hochuli, M., Assoun, M., Ballhausen, D., et al. (2014). Proposed guidelines for the diagnosis and management of methylmalonic and propionic acidemia. Orphanet J. Rare Dis. *9*, 130.

13. Fraser, J.L., and Venditti, C.P. (2016). Methylmalonic and propionic acidemias: Clinical management update. Curr. Opin. Pediatr. *28*, 682–693.

14. Chandler, R.J., Sloan, J., Fu, H., Tsai, M., Stabler, S., Allen, R., Kaestner, K.H., Kazazian, H.H., and Venditti, C.P. (2007). Metabolic phenotype of methylmalonic acidemia in mice and humans: The role of skeletal muscle. BMC Med. Genet. *8*, 64.

15. Kasahara, M., Horikawa, R., Tagawa, M., Uemoto, S., Yokoyama, S., Shibata, Y., Kawano, T., Kuroda, T., Honna, T., Tanaka, K., and Saeki, M. (2006). Current role of liver transplantation for methylmalonic acidemia: A review of the literature. Pediatr. Transplant. *10*, 943–947.

16. Chen, P.W., Hwu, W.L., Ho, M.C., Lee, N.C., Chien, Y.H., Ni, Y.H., and Lee, P.H. (2010). Stabilization of blood methylmalonic acid level in methylmalonic acidemia after liver transplantation. Pediatr. Transplant. *14*, 337–341.

17. Kamei, K., Ito, S., Shigeta, T., Sakamoto, S., Fukuda, A., Horikawa, R., Saito, O., Muguruma, T., Nakagawa, S., Iijima, K., and Kasahara, M. (2011). Preoperative dialysis for liver transplantation in methylmalonic acidemia. Ther. Apher. Dial. *15*, 488–492.

18. Hussein, M.H., Hashimoto, T., Suzuki, T., Daoud, G.A., Goto, T., Nakajima, Y., Kato, T., Hibi, M., Tomishige, H., Hara, F., et al. (2013). Children undergoing liver transplantation for treatment of inherited metabolic diseases are prone to higher oxidative stress, complement activity and transforming growth factor-β1. Ann. Transplant. *18*, 63–68.

19. Niemi, A.K., Kim, I.K., Krueger, C.E., Cowan, T.M., Baugh, N., Farrell, R., Bonham, C.A., Concepcion, W., Esquivel, C.O., and Enns, G.M. (2015). Treatment of methylmalonic acidemia by liver or combined liver-kidney transplantation. J. Pediatr. *166*, 1455–14561.e1.

20. Critelli, K., McKiernan, P., Vockley, J., Mazariegos, G., Squires, R.H., Soltys, K., and Squires, J.E. (2018). Liver transplantation for propionic acidemia and methylmalonic acidemia: Perioperative management and clinical outcomes. Liver Transpl. *24*, 1260–1270.

21. Manoli, I., Sysol, J.R., Li, L., Houillier, P., Garone, C., Wang, C., Zerfas, P.M., Cusmano-Ozog, K., Young, S., Trivedi, N.S., et al. (2013). Targeting proximal tubule mitochondrial dysfunction attenuates the renal disease of methylmalonic acidemia. Proc. Natl. Acad. Sci. USA *110*, 13552–13557.

22. Carrillo-Carrasco, N., Chandler, R.J., Chandrasekaran, S., and Venditti, C.P. (2010). Liver-directed recombinant adeno-associated viral gene delivery rescues a lethal mouse model of methylmalonic acidemia and provides long-term phenotypic correction. Hum. Gene Ther. *21*, 1147–1154.

23. Chandler, R.J., and Venditti, C.P. (2008). Adenovirus-mediated gene delivery rescues a neonatal lethal murine model of mut^0 methylmalonic acidemia. Hum. Gene Ther. *19*, 53–60.

24. Sénac, J.S., Chandler, R.J., Sysol, J.R., Li, L., and Venditti, C.P. (2012). Gene therapy in a murine model of methylmalonic acidemia using rAAV9-mediated gene delivery. Gene Ther. *19*, 385–391.

25. An, D., Schneller, J.L., Frassetto, A., Liang, S., Zhu, X., Park, J.S., Theisen, M., Hong, S.J., Zhou, J., Rajendran, R., et al. (2017). Systemic messenger RNA therapy as a treatment for methylmalonic acidemia. Cell Rep. *21*, 3548–3558.

26. An, D., Frassetto, A., Jacquinet, E., Eybye, M., Milano, J., DeAntonis, C., Nguyen, V., Laureano, R., Milton, J., Sabnis, S., et al. (2019). Long-term efficacy and safety of mRNA therapy in two murine models of methylmalonic acidemia. EBioMedicine *45*, 519–528.

27. Cunningham, S.C., Spinoulas, A., Carpenter, K.H., Wilcken, B., Kuchel, P.W., and Alexander, I.E. (2009). AAV2/8-mediated correction of OTC deficiency is robust in adult but not neonatal Spf^{ash} mice. Mol. Ther. *17*, 1340–1346.

28. Meliani, A., Boisgerault, F., Hardet, R., Marmier, S., Collaud, F., Ronzitti, G., Leborgne, C., Costa Verdera, H., Simon Sola, M., Charles, S., et al. (2018). Antigen-selective modulation of AAV immunogenicity with tolerogenic rapamycin nanoparticles enables successful vector re-administration. Nat. Commun. *9*, 4098.

29. Ilyinskii, P.O., Michaud, A.M., Roy, C.J., Rizzo, G.L., Elkins, S.L., Capela, T., Chowdhury, A.C., Leung, S.S., and Kishimoto, T.K. (2021). Enhancement of liver-directed transgene expression at initial and repeat doses of AAV vectors admixed with ImmTOR nanoparticles. Sci. Adv. *7*, eabd0321.

30. Hösel, M., Huber, A., Bohlen, S., Lucifora, J., Ronzitti, G., Puzzo, F., Boisgerault, F., Hacker, U.T., Kwanten, W.J., Klöting, N., et al. (2017). Autophagy determines efficiency of liver-directed gene therapy with adeno-associated viral vectors. Hepatology *66*, 252–265.

31. Shi, X., Aronson, S.J., Ten Bloemendaal, L., Duijst, S., Bakker, R.S., de Waart, D.R., Bortolussi, G., Collaud, F., Oude Elferink, R.P., Muro, A.F., et al. (2020). Efficacy of AAV8-h*UGT1A1* with rapamycin in neonatal, suckling, and juvenile rats to model treatment in pediatric CNs patients. Mol. Ther. Methods Clin. Dev. *20*, 287–297.

32. Zinn, E., Pacouret, S., Khaychuk, V., Turunen, H.T., Carvalho, L.S., Andres-Mateos, E., Shah, S., Shelke, R., Maurer, A.C., Plovie, E., et al. (2015). In silico reconstruction of the viral evolutionary lineage yields a potent gene therapy vector. Cell Rep. *12*, 1056–1068.

33. Davidoff, A.M., Ng, C.Y.C., Zhou, J., Spence, Y., and Nathwani, A.C. (2003). Sex significantly influences transduction of murine liver by recombinant adeno-associated viral vectors through an androgen-dependent pathway. Blood *102*, 480–488.

34. Pfeifer, C., Aneja, M.K., Hasenpusch, G., and Rudolph, C. (2011). Adeno-associated virus serotype 9-mediated pulmonary transgene expression: Effect of mouse strain, animal gender and lung inflammation. Gene Ther. *18*, 1034–1042.

35. Chai, Z., Zhang, X., Dobbins, A.L., Rigsbee, K.M., Wang, B., Samulski, R.J., and Li, C. (2019). Optimization of dexamethasone administration for maintaining global transduction efficacy of adeno-associated virus serotype 9. Hum. Gene Ther. *30*, 829–840.

36. Chandler, R.J., Zerfas, P.M., Shanske, S., Sloan, J., Hoffmann, V., DiMauro, S., and Venditti, C.P. (2009). Mitochondrial dysfunction in *mut* methylmalonic acidemia. FASEB J. *23*, 1252–1261.

37. Mendell, J.R., Al-Zaidy, S., Shell, R., Arnold, W.D., Rodino-Klapac, L.R., Prior, T.W., Lowes, L., Alfano, L., Berry, K., Church, K., et al. (2017). Single-dose gene-replacement therapy for spinal muscular atrophy. N. Engl. J. Med. *377*, 1713–1722.

38. High, K.A., and Roncarolo, M.G. (2019). Gene therapy. N. Engl. J. Med. *381*, 455–464.

39. Keeler, A.M., and Flotte, T.R. (2019). Recombinant adeno-associated virus gene therapy in light of Luxturna (and Zolgensma and Glybera): Where are we, and how did we get here? Annu. Rev. Virol. *6*, 601–621.

40. Mingozzi, F., and High, K.A. (2017). Overcoming the host immune response to adeno-associated virus gene delivery vectors: The race between clearance, tolerance, neutralization, and escape. Annu. Rev. Virol. *4*, 511–534.

41. Maldonado, R.A., LaMothe, R.A., Ferrari, J.D., Zhang, A.H., Rossi, R.J., Kolte, P.N., Griset, A.P., O'Neil, C., Altreuter, D.H., Browning, E., et al. (2015). Polymeric synthetic nanoparticles for the induction of antigen-specific immunological tolerance. Proc. Natl. Acad. Sci. USA *112*, E156–E165.

42. Kishimoto, T.K., Ferrari, J.D., LaMothe, R.A., Kolte, P.N., Griset, A.P., O'Neil, C., Chan, V., Browning, E., Chalishazar, A., Kuhlman, W., et al. (2016). Improving the efficacy and safety of biologic drugs with tolerogenic nanoparticles. Nat. Nanotechnol. *11*, 890–899.

43. Zhang, A.-H., Rossi, R.J., Yoon, J., Wang, H., and Scott, D.W. (2016). Tolerogenic nanoparticles to induce immunologic tolerance: Prevention and reversal of FVIII inhibitor formation. Cell. Immunol. *301*, 74–81.

44. Lim, H.H., Yi, H., Kishimoto, T.K., Gao, F., Sun, B., and Kishnani, P.S. (2017). A pilot study on using rapamycin-carrying synthetic vaccine particles (SVP) in conjunction with enzyme replacement therapy to induce immune tolerance in Pompe disease. Mol. Genet. Metab. Rep. *13*, 18–22.

45. Mazor, R., King, E.M., Onda, M., Cuburu, N., Addissie, S., Crown, D., Liu, X.F., Kishimoto, T.K., and Pastan, I. (2018). Tolerogenic nanoparticles restore the antitumor activity of recombinant immunotoxins by mitigating immunogenicity. Proc. Natl. Acad. Sci. USA *115*, E733–E742.

46. Perez, B.A., Shutterly, A., Chan, Y.K., Byrne, B.J., and Corti, M. (2020). Management of neuroinflammatory responses to AAV-mediated gene therapies for neurodegenerative diseases. Brain Sci. *10*, E119.

47. Kruzik, A., Fetahagic, D., Hartlieb, B., Dorn, S., Koppensteiner, H., Horling, F.M., Scheiflinger, F., Reipert, B.M., and de la Rosa, M. (2019). Prevalence of anti-adeno-associated virus immune responses in international cohorts of healthy donors. Mol. Ther. Methods Clin. Dev. *14*, 126–133.

48. Majowicz, A., Nijmeijer, B., Lampen, M.H., Spronck, L., de Haan, M., Petry, H., van Deventer, S.J., Meyer, C., Tangelder, M., and Ferreira, V. (2019). Therapeutic hFIX activity achieved after single AAV5-hFIX treatment in hemophilia B patients and NHPs with pre-existing anti-AAV5 NABs. Mol. Ther. Methods Clin. Dev. *14*, 27–36.

49. George, L.A., Ragni, M.V., Rasko, J.E.J., Raffini, L.J., Samelson-Jones, B.J., Ozelo, M., Hazbon, M., Runowski, A.R., Wellman, J.A., Wachtel, K., et al. (2020). Long-term follow-up of the first in human intravascular delivery of AAV for gene transfer: AAV2-hFIX16 for severe hemophilia B. Mol. Ther. *28*, 2073–2082.

50. Manning, W.C., Zhou, S., Bland, M.P., Escobedo, J.A., and Dwarki, V. (1998). Transient immunosuppression allows transgene expression following readministration of adeno-associated viral vectors. Hum. Gene Ther. *9*, 477–485.

51. Mingozzi, F., Chen, Y., Murphy, S.L., Edmonson, S.C., Tai, A., Price, S.D., Metzger, M.E., Zhou, S., Wright, J.F., Donahue, R.E., et al. (2012). Pharmacological modulation of humoral immunity in a nonhuman primate model of AAV gene transfer for hemophilia B. Mol. Ther. *20*, 1410–1416.

52. Jiang, H., Couto, L.B., Patarroyo-White, S., Liu, T., Nagy, D., Vargas, J.A., Zhou, S., Scallan, C.D., Sommer, J., Vijay, S., et al. (2006). Effects of transient immunosuppression on adenoassociated, virus-mediated, liver-directed gene transfer in rhesus macaques and implications for human gene therapy. Blood *108*, 3321–3328.

53. Mingozzi, F., Anguela, X.M., Pavani, G., Chen, Y., Davidson, R.J., Hui, D.J., Yazicioglu, M., Elkouby, L., Hinderer, C.J., Faella, A., et al. (2013). Overcoming preexisting humoral immunity to AAV using capsid decoys. Sci. Transl. Med. *5*, 194ra92.

54. Monteilhet, V., Saheb, S., Boutin, S., Leborgne, C., Veron, P., Montus, M.-F., Moullier, P., Benveniste, O., and Masurier, C. (2011). A 10 patient case report on the impact of plasmapheresis upon neutralizing factors against adeno-associated virus (AAV) types 1, 2, 6, and 8. Mol. Ther. *19*, 2084–2091.

55. Chicoine, L.G., Montgomery, C.L., Bremer, W.G., Shontz, K.M., Griffin, D.A., Heller, K.N., Lewis, S., Malik, V., Grose, W.E., Shilling, C.J., et al. (2014). Plasmapheresis eliminates the negative impact of AAV antibodies on microdystrophin gene expression following vascular delivery. Mol. Ther. *22*, 338–347.

56. Salas, D., Kwikkers, K.L., Zabaleta, N., Bazo, A., Petry, H., van Deventer, S.J., Aseguinolaza, G.G., and Ferreira, V. (2019). Immunoadsorption enables successful rAAV5-mediated repeated hepatic gene delivery in nonhuman primates. Blood Adv. *3*, 2632–2641.

57. Bertin, B., Veron, P., Leborgne, C., Deschamps, J.Y., Moullec, S., Fromes, Y., Collaud, F., Boutin, S., Latournerie, V., van Wittenberghe, L., et al. (2020). Capsid-specific removal of circulating antibodies to adeno-associated virus vectors. Sci. Rep. *10*, 864.

58. Orlowski, A., Katz, M.G., Gubara, S.M., Fargnoli, A.S., Fish, K.M., and Weber, T. (2020). Successful transduction with AAV vectors after selective depletion of anti-AAV antibodies by immunoadsorption. Mol. Ther. Methods Clin. Dev. *16*, 192–203.

59. Leborgne, C., Barbon, E., Alexander, J.M., Hanby, H., Delignat, S., Cohen, D.M., Collaud, F., Muraleetharan, S., Lupo, D., Silverberg, J., et al. (2020). IgG-cleaving endopeptidase enables in vivo gene therapy in the presence of anti-AAV neutralizing antibodies. Nat. Med. *26*, 1096–1101.

60. Mingozzi, F., Hasbrouck, N.C., Basner-Tschakarjan, E., Edmonson, S.A., Hui, D.J., Sabatino, D.E., Zhou, S., Wright, J.F., Jiang, H., Pierce, G.F., et al. (2007). Modulation of tolerance to the transgene product in a nonhuman primate model of AAV-mediated gene transfer to liver. Blood *110*, 2334–2341.

61. Luciani, A., Schumann, A., Berquez, M., Chen, Z., Nieri, D., Failli, M., Debaix, H., Festa, B.P., Tokonami, N., Raimondi, A., et al. (2020). Impaired mitophagy links mitochondrial disease to epithelial stress in methylmalonyl-CoA mutase deficiency. Nat. Commun. *11*, 970.

62. Chandler, R.J., LaFave, M.C., Varshney, G.K., Trivedi, N.S., Carrillo-Carrasco, N., Senac, J.S., Wu, W., Hoffmann, V., Elkahloun, A.G., Burgess, S.M., and Venditti, C.P. (2015). Vector design influences hepatic genotoxicity after adeno-associated virus gene therapy. J. Clin. Invest. *125*, 870–880.

OMTM, Volume *22*

Supplemental information

ImmTOR nanoparticles enhance AAV transgene expression after initial and repeat dosing in a mouse model of methylmalonic acidemia

Petr O. Ilyinskii, Alicia M. Michaud, Gina L. Rizzo, Christopher J. Roy, Sheldon S. Leung, Stephanie L. Elkins, Teresa Capela, Aparajita Chowdhury, Lina Li, Randy J. Chandler, Irini Manoli, Eva Andres-Mateos, Lloyd P.M. Johnston, Luk H. Vandenberghe, Charles P. Venditti, and Takashi Kei Kishimoto

Supplementary Materials

Supplementary Table S1. Absolute levels of plasma MMA (pMMA) at 14 days after treatment with Anc80-MMUT, ImmTOR and dexamethasone (Dex-P). Averages ± SD are shown for each group. Number of subjects in each group is shown in parentheses.

Treatment	Anc80-MMUT (15)	Anc80-MMUT (15)	Anc80-MMUT (12)	Anc80-MMUT (7)	Anc80-MMUT (7)
AAV	2.5E12 vg/kg	2.5E12 vg/kg	2.5E12 vg/kg	2.5E12 vg/kg	2.5E12 vg/kg
ImmTOR	None	100 µg	300 µg	None	300 µg
Dexp-P	None	None	None	10 mg/kg	10 mg/kg
pMMA	228 ± 98 µM	127 ± 39 µM	159 ± 80 µM	246 ± 107 µM	100 ± 23 µM

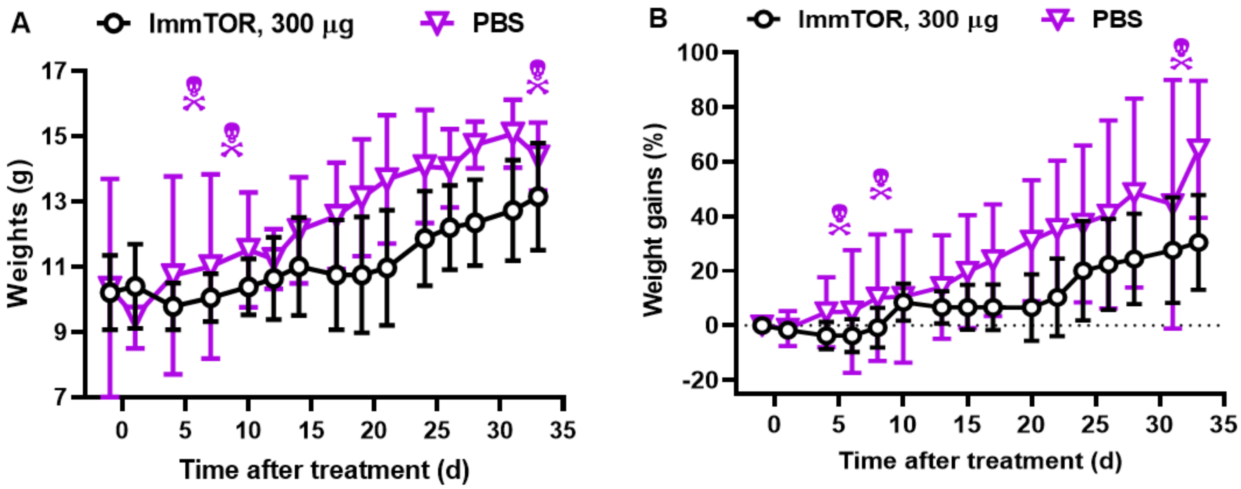

Supplementary Figure S1. Weight gains in MCK mice treated with ImmTOR or mock-treated. Absolute weights (**A**) or weight gains in % vs. pre-injection weight (**B**) are shown with times of mouse deaths in mock-treated group indicated by crossbones sign.

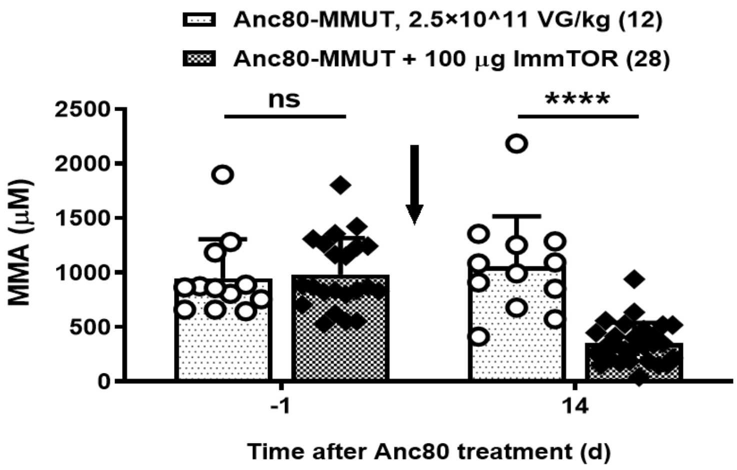

Supplementary Figure S2. ImmTOR provides added benefit at subtherapeutic doses of Anc80-MMUT. *Mmut*$^{-/-}$*;* Tg$^{\text{INS-MCK-}\textit{Mmut}}$ mice at 14 days of age were treated once with Anc80-MMUT (2.5×10^{11} vg/kg) either alone or combined with ImmTOR (100 µg). Plasma methylmalonic acid (pMMA) levels before the treatment (day -1) and at 14 days after a single Anc80-MMUT administration are shown with numbers of mice in each group indicated in parentheses. Statistical significance is indicated (ns – not significant, **** – $p < 0.0001$).

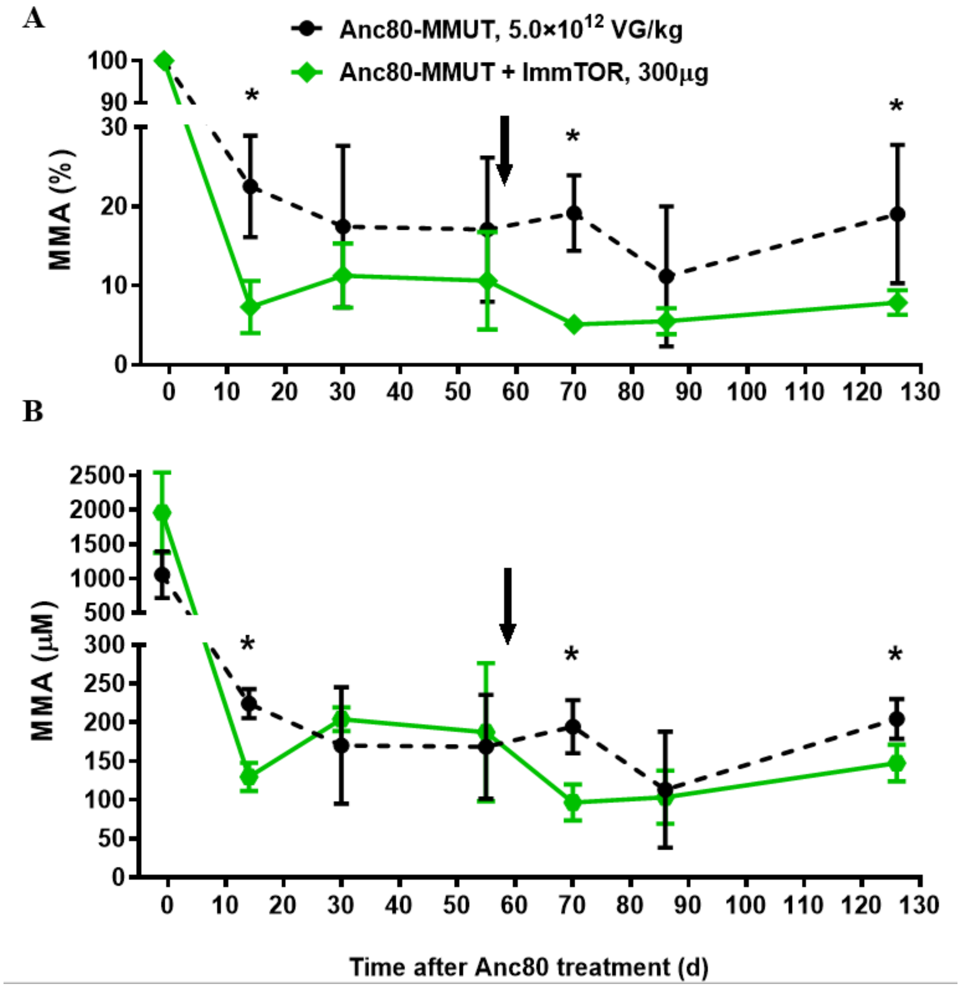

Supplementary Figure S3. Co-administration of high therapeutic dose of Anc80-MMUT with ImmTOR elevates transgene activity. Anc80-MMUT (5.0×10^{12} vg/kg) alone or combined with 300 µg of ImmTOR was injected twice on days 0 and 56 in groups of 3-4 mice. Plasma MMA levels are shown for the duration of the study (126 days after initial treatment) as % vs. pre-treatment levels as 100% (**A**) or in absolute numbers (**B**). Time-points with statistically significant (* – $p<0.05$) differences vs. group not receiving ImmTOR are indicated. Day 56 repeat injection is indicated by arrow. Averages with SD are shown.

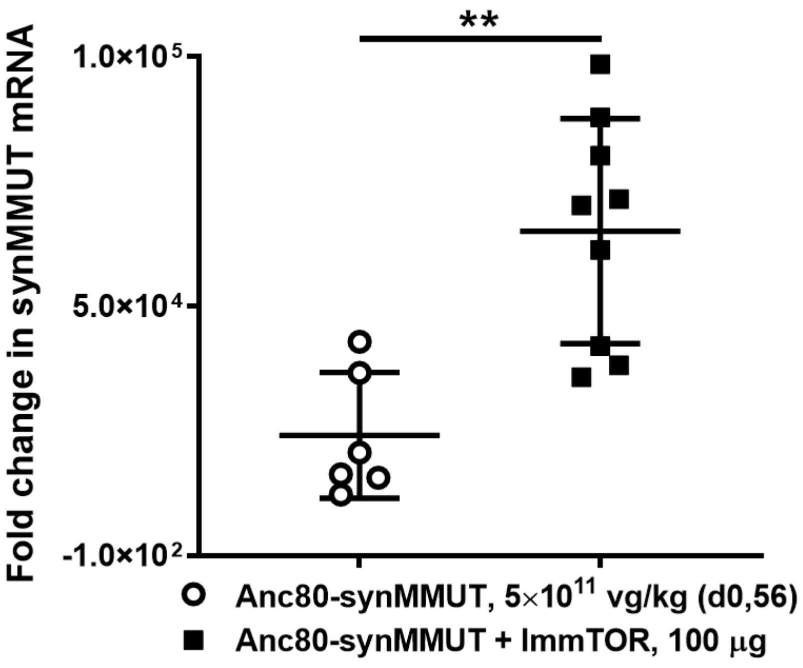

Supplementary Figure S4. AAV-driven *MMUT* mRNA expression after repeated injections with Anc80-MMUT combined with ImmTOR. *synMMUT* mRNA levels in livers of wild-type C57BL/6 female mice after two (days 0 and 56) injections with Anc80-synMMUT with or without 100 µg of ImmTOR as measured by fold increase (GAPDH-normalized) over untreated baseline 43 days after repeat vector dose (day 99 after initial treatment). Statistical significance (** – $p<0.01$) is shown. Averages with SD are shown.

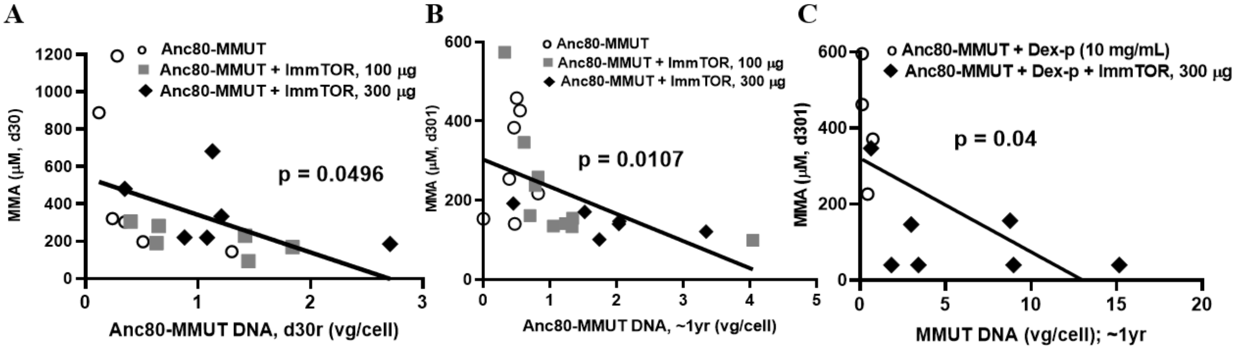

Supplementary Figure S5. Inverse correlation of short-term and long-term AAV transduction efficacy and serum levels of methylmalonic acid in individual studies. **A.** Viral DNA copy numbers and serum levels of methylmalonic acid have been measured on day 30 following a single injection with Anc80-MMUT alone or combined with 100 or 300 µg ImmTOR. Data shown in Figs. 1B and 2A are used for analysis. **B, C.** Viral DNA copy numbers and serum levels of methylmalonic acid have been measured 1 year after two doses of Anc80-MMUT administered alone or combined with 100 or 300 µg ImmTOR. Data from Figs. 4A and 4C (**B**) or from Figs. 5A and 5C (**C**) are used for analysis. P value of correlation is shown for each graph.

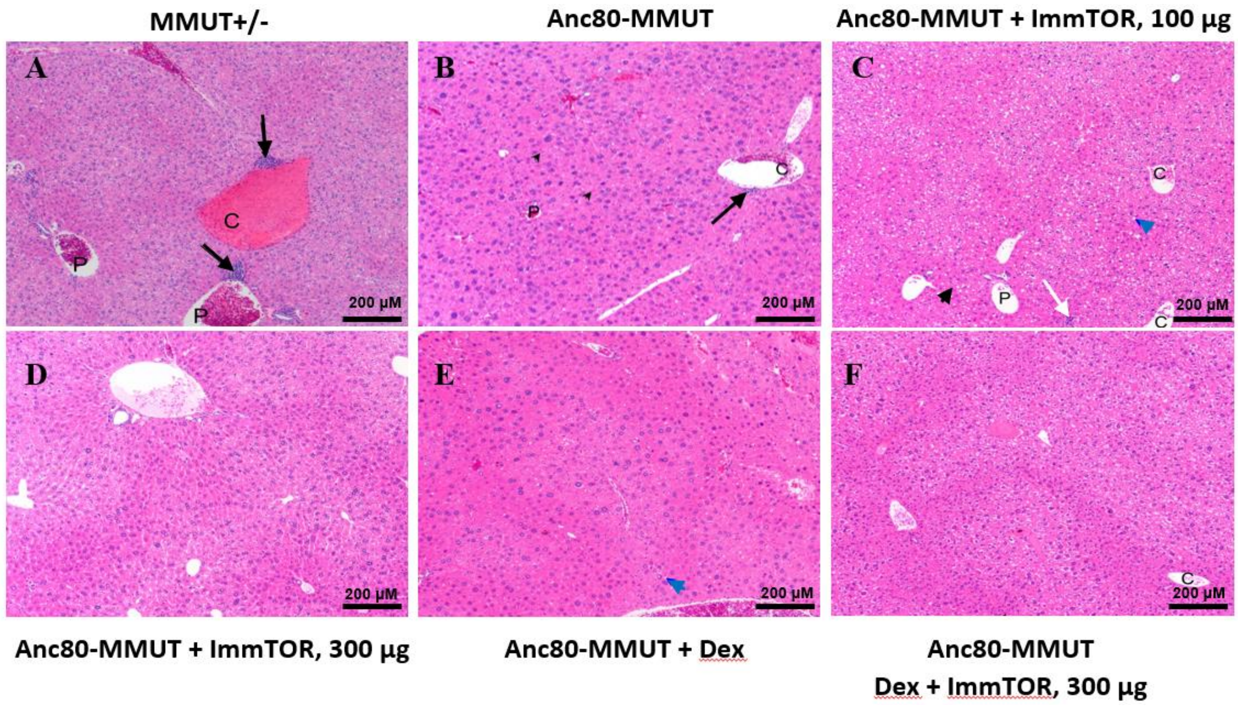

Supplementary Figure S6. Long-term alleviation of hepatic pathology by Anc80-MMUT and ImmTOR. Representative photomicrographs of liver sections from heterozygous *Mmut*$^{+/-}$ (**A**),

and Anc80-MMUT-treated *Mmut*$^{-/-}$; Tg$^{\text{INS-MCK-}\textit{Mmut}}$ mice (**B-F**) are shown (40×). Anc80-MMUT (days 0 and 56) was used alone (B) or combined with 100 µg (**C**) or 300 µg (**D**) of ImmTOR, combined with dexamethasone (**E**) or dexamethasone and 300 µg ImmTOR (**F**). Hematoxylin and eosin staining of liver sections from 1-year old mice are shown, scale bars: 200 µm. Heterozygous control (**A**) shows small aggregates of perivascular mononuclear cells (black arrows) adjacent to both portal tracts (P) and central veins (C). Livers from mice treated Anc80-MMUT (**B**, **E**) showed minimal mononuclear cell infiltration (black arrow) accompanied by a few necrotic hepatocytes as well as low numbers of hepatocytes with intracytoplasmic inclusions (black arrowheads) or vacuoles (blue arrowheads). Mice co-treated with low dose ImmTOR (**C**) shown minimal, random mixed cell inflammation (white arrows), hepatocyte vacuolization (blue arrowheads), and hepatocyte intracytoplasmic inclusions (black arrowheads), while in mice co-treated with high dose ImmTOR (**D**, **F**) histologic findings were minimal to absent.

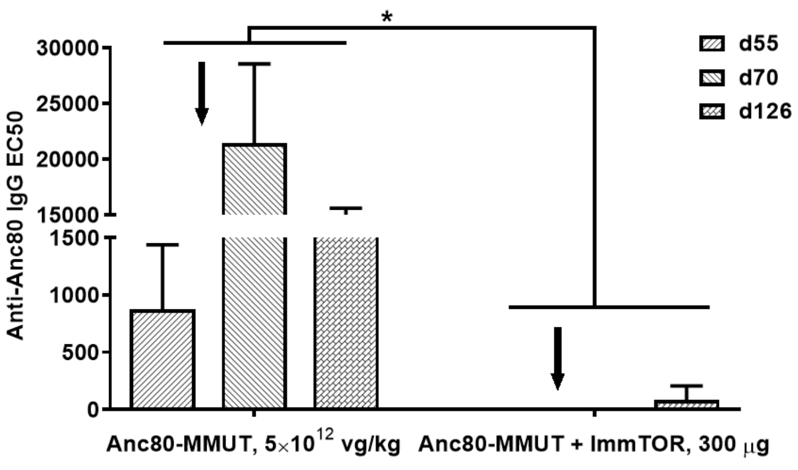

Supplementary Figure S7. Co-administration of high therapeutic dose of Anc80-MMUT with ImmTOR prevents IgG antibody formation. Anc80-MMUT (5.0×10^{12} vg/kg) alone or combined with 300 µg of ImmTOR was injected twice on days 0 and 56 in groups of 3-4 *Mmut*$^{-/-}$

; Tg$^{INS\text{-}MCK\text{-}Mmut}$ mice. EC_{50} of Anc80 IgG in serum was measured at times indicated. Time-points with statistically significant (* – p<0.05) differences between groups are indicated. Day 56 repeat injection is indicated by arrow. Averages with SD are shown.

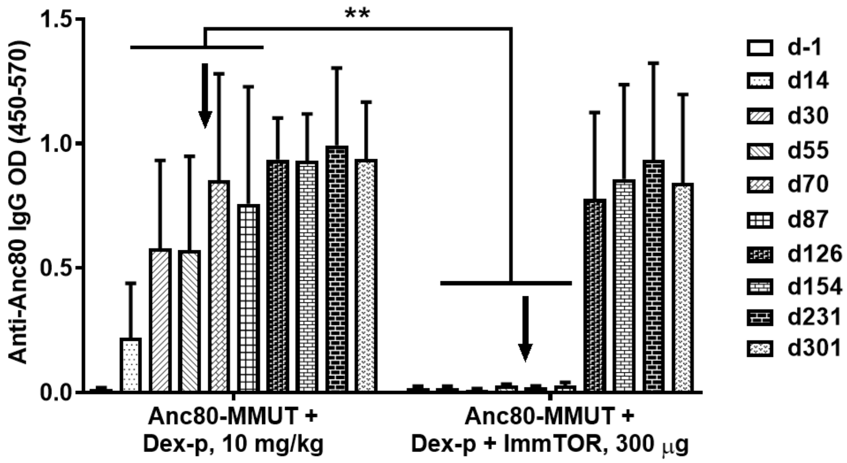

Supplementary Figure S8. Co-administration of high therapeutic dose of Anc80-MMUT with ImmTOR and dexamethasone prevents IgG antibody formation, while dexamethasone alone is ineffective. Anc80-MMUT (2.5×10^{12} vg/kg) alone or combined with 300 µg of ImmTOR was injected twice on days 0 and 56 in groups of 5-7 *Mmut*$^{-/-}$*;* Tg$^{INS\text{-}MCK\text{-}Mmut}$ mice (see Fig. 5). Both groups also received 10 mg/ml dexamethasone concurrently with Anc80 inoculation. Anc80 IgG in serum was measured at times indicated and presented as top OD. Time-points with statistically significant (** – p<0.01) differences between groups receiving and not receiving ImmTOR are indicated. Day 56 repeat injection is indicated by arrow. Averages with SD are shown.
